# Pleiotropic pharmacological activities and multiple-organ toxicities of triptolide: a programmed cell death perspective

**DOI:** 10.1186/s13020-026-01369-1

**Published:** 2026-03-17

**Authors:** Yuan Mao, LiWen Huang, HongPing Long, Qi Huang, Fenghua Kang, Yi-Kun Wang

**Affiliations:** 1 Xiangya School of Pharmaceutical Sciences, Central South University, Changsha, 410013 Hunan China; 2 Center for Medical Research and Innovation, The First Hospital of Hunan University of Chinese Medicine, Changsha, China; 3 Department of Pharmacy, Xiangya Hospital Central South University, Changsha, 410013 Hunan China

**Keywords:** Triptolide, Programmed cell death, Molecular mechanism, Pharmacological activities, Multi-organ toxicities, Nano-delivery system

## Abstract

Triptolide, a bioactive triepoxide diterpenoid extracted from *Tripterygium wilfordii* Hook. f., has demonstrated broad pharmacological activities and significant toxicities. Mechanistically, triptolide has exerted therapeutic effects by regulating programmed cell death (PCD) through multiple pathways; however, its toxic reactions have been closely associated with this process. This review systematically summarizes the molecular mechanisms by which triptolide regulated various forms of PCD and its application progress in disease treatment, including apoptosis, autophagy, pyroptosis, ferroptosis, cuproptosis, necroptosis, and PANoptosis. A plethora of extant studies have revealed that triptolide exerted a regulatory effect on the PCD networks by intervening in multiple important signaling pathways and their key signaling nodes. Nevertheless, due to its poor target specificity, triptolide has resulted in multi-organ toxicities, which has in turn limited its clinical translation. Nano-delivery systems have explored as a potential strategy to mitigate the toxicity and improve the efficacy of triptolide by enhancing its tissue targeting (Specific nanocarriers can increase LD_50_ from 0.48 to 0.88 mg/kg). Furthermore, key pharmacodynamic evidences from triptolide’s current clinical studies are limited, necessitating the elucidation of precise target sites and the advancement of standardized clinical trials. This review systematically integrates the pleiotropic pharmacological activities and multiple-organ toxicities of triptolide from a PCD perspective, providing novel insights and theoretical references for overcoming its clinical translation barriers.

**Graphical Abstract:**

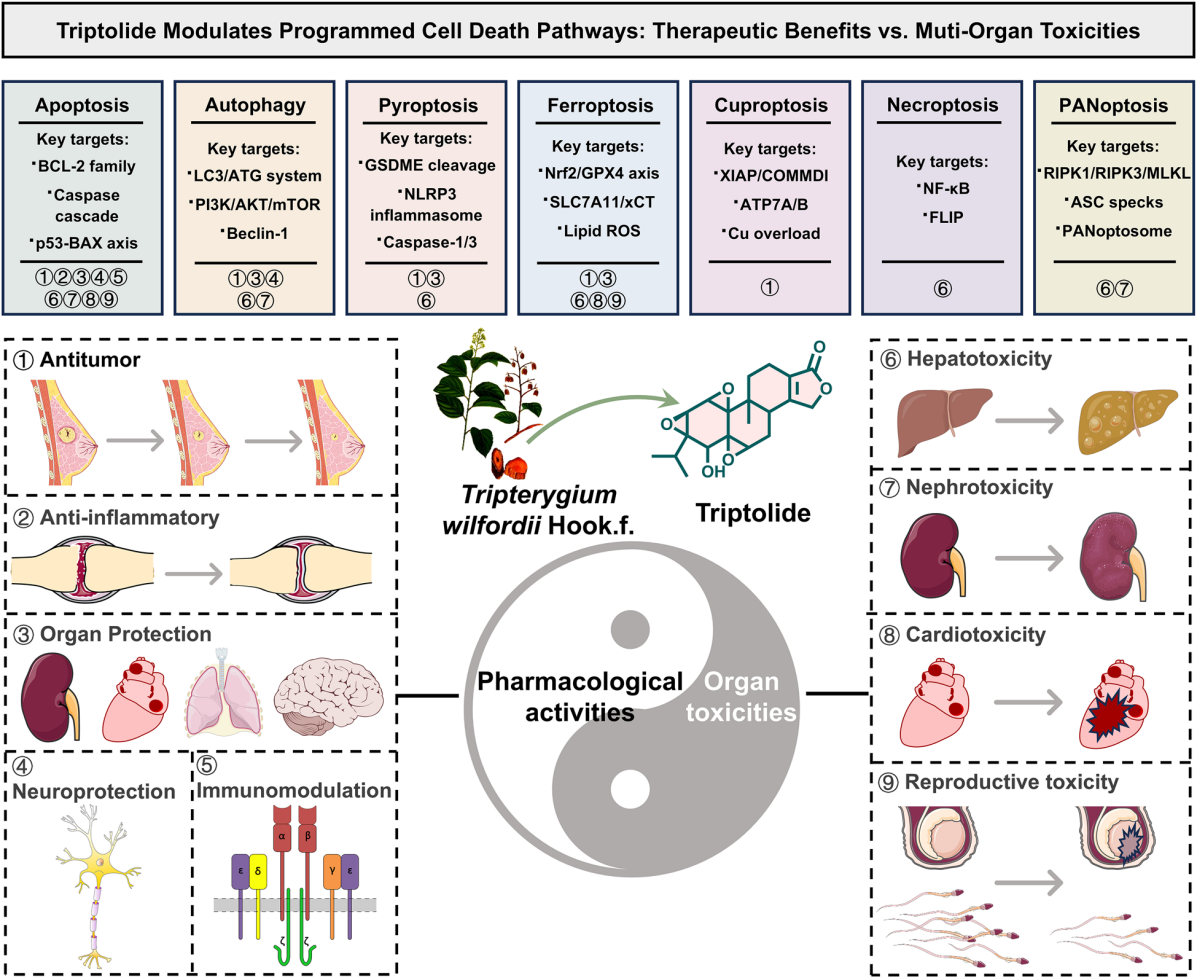

## Introduction

“To combat poison with poison” is special Traditional Chinese Medicine Theory, toxic herb *Tripterygium wilfordii* Hook.f. has long been regarded as a representative sample of this theory [1]. Triptolide is the principal bioactive constituent of three medicinal *Tripterygium* species (*T. wilfordii*, *T. regelii* Sprague & Takeda, and *T. hypoglaucum* (H.Lév.) Hutch.) [2, 3], and its multi-effect pharmacology highly aligned with “to combat poison with poison” principle of *T. wilfordii*, displaying potent anti-inflammatory, immunosuppressive and antitumor activities [4–9]. Valuably, triptolide has been listed by *Cell* as one of the most five promising natural lead compounds with potential for development of modern drug, due to its multifunctional pharmacological properties at nanomolar concentrations [10]. However, the therapeutic window of triptolide substantially narrow (with a mouse LD_50_ of 0.83 mg/kg) [11], especially triptolide has induced toxicities in the liver, kidneys, heart, and reproductive organs [12]. From a dialectical perspective, the toxicity of triptolide and *T. wilfordii* can manifest as cytotoxicity against tumor cells and inflammatory cells in pharmacology, and as damage to normal cells and organs in toxicology.

Mechanically, the modulation of various programmed cell death (PCD) pathways by triptolide may associated with for its dual attributes of therapy and toxicity, due to its unique epoxide structure and multi-target regulatory capacity [13]. The “to combat poison with poison” effect of triptolide may achieve through the potent induction of various forms of PCD, including but not limited to apoptosis, autophagy, and pyroptosis [13]. Triptolide-mediated-PCD has enabled the specific elimination of pathological cells such as tumor cells and abnormally activated immune cells, representing a central mechanism underlying its pharmacological action [14]. Notably, triptolide has exhibited poor target specificity in regulating PCD. Although inducing death in pathological cells, triptolide may also trigger similar PCD processes in normal tissue cells, ultimately leading to dose-dependent multiple-organs damage, which has constituted the significant source of triptolide’s toxicity [15, 16].

The efficacious-poison duality of *Tripterygium* herbs and triptolide may closely associated with bidirectional regulation of PCD. Dialectically, the relationship between efficacy and toxicity in *Tripterygium* herbs has been not a simple dichotomy, but rather a dynamic unity that has been interdependent and mutually transformative within the context of specific diseases. The patterns of transformation must be understood within the “person-medicine-disease” system. In clinical practice, only by adhering to “differentiated diagnosis-based medication, precise dosing, and rational drug combinations” can the “toxic properties” of *Tripterygium* herbs be transformed into “therapeutic efficacy,” ultimately achieving the health goal of “yin-yang balance” [12]. *Tripterygium* herbs and triptolide has served as an effective remedy through precise regulation of PCD, whereas disordered or excessive PCD has underlain their potential toxicity [14]. From this perspective, the principle of “to combat poison with poison” can be reinterpreted as a primitive form of targeted intervention against abnormal cell fate [13]. Therefore, exploring bidirectional regulation mechanism of PCD of triptolide will help bridge empirical traditional *Tripterygium* herb medicine and modern precision medicine, providing a rational mechanistic basis for the safe and effective application of *Tripterygium* herbs.

In this review, the molecular mechanisms underlying the pharmacological activities and multi-organ toxicities of triptolide by regulating various PCD were summarized, majorly including apoptosis, autophagy, pyroptosis, ferroptosis, cuproptosis, necroptosis, and PANoptosis, and provides an in-depth exploration of triptolide in signal pathway regulation, intervention of key targets, and determination of cellular fate. To overcome the key limitations of triptolide, such as severe off-target toxicities, poor delivery and tissue selectivity, advanced nano-delivery systems has represented a highly promising strategy, mitigating off-target toxicity and boosting therapeutic efficacy. Furthermore, the clinical tests and evidence of triptolide formulations or derivatives are limited, both elucidating triptolide’s core target molecules and integrated signaling networks should be strengthened in the future to establish a more robust foundation for the successful translation of triptolide derivative drugs. This review provides a valuable reference for guiding clinical trials and future clinical applications of triptolide.

## Literature retrieval

**Fig. 1:**
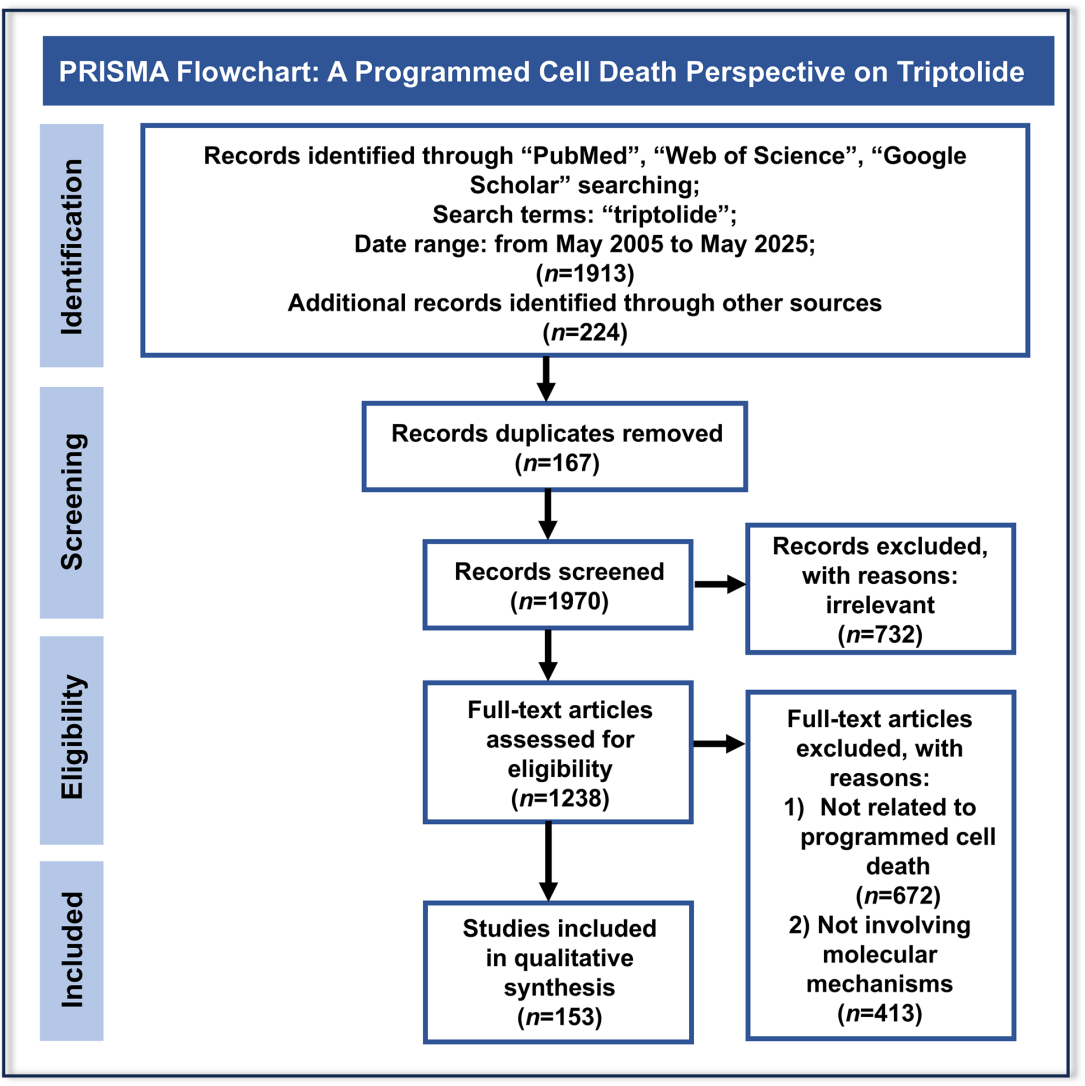
PRISMA flow diagram for the systematic review on Pleiotropic pharmacological activities and multiple-organ toxicities of triptolide: a programmed cell death perspective

A comprehensive literature search was conducted in multiple scientific databases, included Web of Science, PubMed, and Google Scholar, covering publications from May 2005 to May 2025 using the search term “triptolide”. A total of 153 relevant studies on triptolide’s pleiotropic pharmacological activities and multi-organ toxicities were systematically collected, screened, and synthesized from the perspective of programmed cell death, adhering to predefined inclusion and exclusion criteria (Fig. 1).

## Bidirectional apoptosis modulation by triptolide generates pharmacological effects and toxicities

**Fig. 2:**
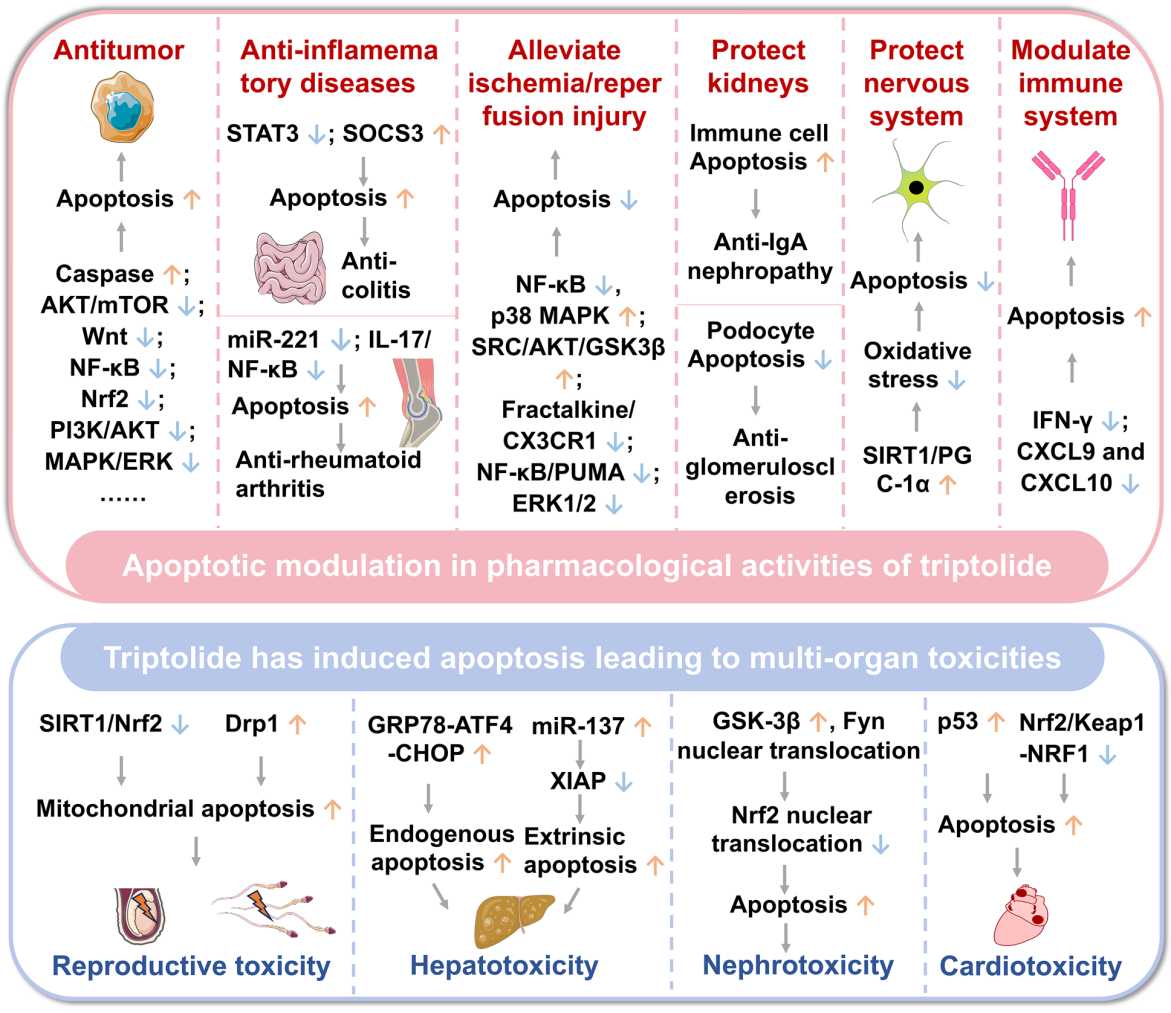
Bidirectional apoptosis modulation by triptolide generates pharmacological effects and toxicities. ↑: Activation or Upregulation; ↓: Inhibition or Downregulation. Other gene and protein abbreviations appearing in this figure can be found in the Abbreviation List

Apoptosis plays a key role in maintaining tissue homeostasis and regulating disease progression and drug effects (activities and toxicities) [17]. Triptolide has displayed significant therapeutic potential by targeting multiple apoptotic molecules and pathways, showing efficacy in various pathological conditions, including cancer, ischemia/reperfusion injury, inflammatory diseases, and liver and kidney diseases [18–21]. However, it has also been reported to cause toxicities in hepatic, renal, cardiac and testicular tissues [22–24] (Fig. 2).

### Apoptotic modulation in pharmacological activities of triptolide

#### Promoting apoptosis to exert antitumor effects

Triptolide has been demonstrated to promote tumor cell apoptosis via the mediation of endoplasmic reticulum stress in intrinsic apoptosis pathway, exhibiting significant cytotoxic effects in various cancers [19, 25–33]. 300 μM triptolide elevated reactive oxygen species (ROS) levels and induced endoplasmic reticulum stress and apoptosis by targeting peroxiredoxin 2 in human gastric cancer cell lines AGS and IM95, providing a new insight into the potential mechanisms of triptolide for treating gastric cancer [34]. The combination of 1–15 ng/mL triptolide with cisplatin enhanced cisplatin-induced mitochondrial apoptosis in gastric cancer SC-M1 cells, as evidenced by the activation of Caspase-3, Caspase-9, and Poly(ADP-ribose) Polymerase (PARP) cleavage. This effect was further confirmed in a severe combined immunodeficiency mouse xenograft model, supporting the potential clinical application of triptolide-cisplatin combination therapy [35].

Targeting mitochondria, triptolide has promoted endogenous apoptosis by regulating the B-cell lymphoma 2 (BCL-2) protein family, demonstrating significant antitumor activity in various types of leukemia. In the context of acute myeloid leukemia (AML), the combination of triptolide with the BCL-2 inhibitor ABT-199 led to the downregulation of Myeloid cell leukemia 1 (MCL-1) and upregulation of pro-apoptotic BH3-only proteins, resulting in mitochondrial apoptosis pathway activation, effectively overcoming chemotherapy resistance and prolonging survival in xenograft animal models [36]. In the treatment of chronic myeloid leukemia (CML), triptolide was observed to effectively downregulate Bcr-Abl mRNA and protein levels independently of the caspase or proteasome pathways and then induced mitochondrial-dependent apoptosis in primary CML cells with Bcr-Abl-T315I mutations and STI571 resistance, suggesting that triptolide represented a promising therapeutic agent for overcoming STI571 resistance in CML cells [37]. In the absence of p53 and with unaltered BCL2-Associated X Protein (BAX) levels, 20 nM triptolide still triggered caspase-mediated BCL-2 cleavage, cytochrome C release, and amplification of the caspase cascade in human promyelocytic leukemia HL-60 cell lines, leading to mitochondrial-dependent apoptosis and exerting a therapeutic effect on promyelocytic leukemia [38].

In addition to regulating endogenous apoptosis, triptolide has been shown to exert anti-AML effects by activating the extrinsic apoptosis pathway and modulating CFLAR protein expression. Gene expression analysis in 60 European AML patients demonstrated that triptolide significantly upregulated apoptosis-related genes, including cellular FLICE-like inhibitory protein (*CFLAR*), peptidylprolyl isomerase like 3 (*PPIL3*), caspase 8 (*CASP8*), caspase 10 (*CASP10*), and signal transducer and activator of transcription 6 (*STAT6*). Mechanistically, *CFLAR* knockdown markedly enhanced sensitivity to triptolide in Human Acute Monocytic Leukemia cell line-1 AML cells, implying *CFLAR* as a key target of triptolide’s cytotoxic effects [39].

Triptolide has also exhibited multidimensional antitumor effects in non-small cell lung cancer. The mechanism by which it promoted apoptosis involved a reduction in 6-Phosphofructo-2-kinase/Fructose-2,6-bisphosphatase 2 (PFKFB2) expression, resulting in the inhibition of phosphatidylinositol 3-kinase (PI3K)/AKT serine/threonine kinase (AKT) pathway [40]; the blocking of interleukin-6 (IL-6)/signal transducer and activator of transcription 3 (STAT3) axis activation [41]; the interference with Wingless/Integrated (Wnt) pathway transmission [42]; the inhibition of AKT/mechanistic target of rapamycin (mTOR)/hexokinase II (HKII) network [43]; and the activation of calcium/calmodulin-dependent protein kinase kinase beta (CaMKKβ)-AMP-activated protein kinase (AMPK) pathway [44]. Furthermore, triptolide has been shown to selectively inhibit the activity of nucleotide excision repair, thus inducing apoptosis in lung cancer cells and enhancing the efficacy of cisplatin treatment in these cells [45].

Triptolide effectively modulated nuclear factor kappa-light-chain-enhancer of activated B cells (NF-κB), mitogen-activated protein kinase (MAPK)/extracellular signal-regulated kinase (ERK), and PI3K/AKT pathways, promoting tumor cell apoptosis and exhibiting significant antitumor activity across a range of cancer models (Table 1). This majorly included nasopharyngeal carcinoma [26], adrenocorticotropic hormone-secreting pituitary adenoma [19], osteosarcoma [27], thyroid cancer [28, 29], breast cancer [46], glioma [47], Burkitt lymphoma [48], multiple myeloma [20, 49], cervical cancer [50], prostate cancer [51], esophageal squamous cell carcinoma [30, 31], bladder cancer [32, 33], and renal cell carcinoma [52].

**Table 1:** Triptolide exerts antitumor activity by promoting apoptosis

Cancer types	Cell lines/models	Dosage	Molecular mechanisms	Refs.
NSCLC	Human lung cancer cell line PC-9	10–50 nM TP, in vitro	TP targeted miR-21, by ↑PTEN	[177]
NSCLC	A549 and H1299 cells	50–150 nM, in vitro	↓PFKFB2 led to↑PI3K/AKT	[40]
NSCLC	Mouse xenograft model	0.8 mg/kg TP, s.c	↓PFKFB2 expression led to ↑PI3K/AKT	[40]
NSCLC	PC9 and A549 cells	30–120 nM TP, in vitro	↓IL-6/STAT3 axis	[41]
NSCLC	A549, H460, H358, and H1299 cell lines	1–10 nM TP, in vitro	↓Wnt signaling	[42]
NSCLC	Mouse xenograft model	1.5 mg/kg TP, s.c	↓Wnt signaling	[42]
NSCLC	H1299 andNCI-H460 cell lines	50–150 nM TP, in vitro	↓AKT/mTOR HKII	[43]
Lung cancer	HTB182, A549, CRL5810, and CRL5922 lung cancer cells	5 or 10 ng/mL TP, in vitro	↓Nucleotide excision repair	[45]
Gastric cancer	Gastric cancer SC-M1 cells	1–15 ng/mL TP, in vitro	↑Caspase-3 and Caspase-9, and PARP was cleaved in SC-M1 cells	[35]
Gastric cancer	Severe combined immunodeficiency mouse xenograft	0.4 mg/kg TP + 3 mg/kg Cisplatin, s.c	↑Caspase-3 and Caspase-9, and PARP was cleaved in SC-M1 cells	[35]
Gastric cancer	Human gastric cancer cell lines AGS and IM95	300 μM TP, in vitro	↑ROS, resulting in endoplasmic reticulum stress	[34]
AML	MV4-11 and MOLM-13 cells	1.25–5 nM TP + ABT-199, in vitro	↓MCL-1, and↑pro-apoptotic BH3 pure proteins	[36]
AML	Xenotransplantation model	0.5 mg/kg TP, i.p. + 50 mg/kg ABT-199, i.g	↓MCL-1, and↑pro-apoptotic BH3 pure proteins	[36]
AML	OCI-AML3, U937, and Jurkat cells	–	↓XIAP and ↑p53-mediated DR5	[25]
AML	60 European AML patients and THP1 AML cell line	–	↑Genes related to apoptosis (*CFLAR*, *PPIL3*, *CASP-8*, *CASP-10*, and *STAT6*)	[39]
Promyelocytic leukemia	HL-60 and MCF-7 cell lines	20 nM TP, in vitro	↑BCL-2 cleavage, mitochondrial cytochrome C release, and Caspase	[38]
CML	CML cell lines and primary cells from CML patients clinically resistant to STI571	5–50 nM TP, in vitro	↓Bcr-Abl transcription, and ↓survivin, MCL-1, and AKT	[37]
Bladder cancer	T24R2 cells	–	↑Caspase-3, 8, and 9, PARP, and cytochrome C	[32]
Bladder cancer	Bladder cancer cell lines EJ and UMUC3	72 nM TP + 1.9 μg/mL Gisithamine, or 50 nM TP + 0.25 μg/mL Gisithamine, in vitro	↑Caspase-8 and BCL-XL proteins, ↑ROS, and ↓AKT/GSK3β pathway	[33]
Nasopharyngeal carcinoma	Human nasopharyngeal carcinoma cells	2–8 Gy IR + 2–8 ng/mL TP, in vitro	↑BAX expression and ↓NF-κB p65 phosphorylation	[26]
Nasopharyngeal carcinoma	Nasopharyngeal carcinoma cell line HONE-1	5–50 nM TP, in vitro	↓*Lnc-THOR-IGF2BP1* signaling	[178]
Adrenocorticotropic hormone-secreting pituitary adenoma	AtT-20 cell line	25-400nM TP, in vitro	↑Mitochondrial membrane depolarization, ↑Caspase-3, ↓BCL2/BAX ratio, and ↓phosphorylation of NF-κB p65 subunit and extracellular ERK	[19]
Adrenocorticotropic hormone-secreting pituitary adenoma	Xenotransplantation in mice	0.15 mg/kg TP, i.p	↑Mitochondrial membrane depolarization, ↑Caspase-3, ↓BCL2/BAX ratio, and↓phosphorylation of NF-κB p65 subunit and extracellular ERK	[19]
Osteosarcoma	Human osteosarcoma U2OS cells	100 nM TP + 30 μM AMD3100, in vitro	↓The phosphorylation levels of ERK1/2, AKT, STAT3, ↓the nuclear translocation and phosphorylation of NF-κB p65	[27]
Osteosarcoma	MG-63 cells	50–200 nM TP, in vitro	↑TRAIL-DR-5 pathway	[179]
Thyroid cancer	Human ATC cell line TA-K cells	–	↓NF-κB independently of p53	[28]
Thyroid cancer	Thyroid cancer cell line TPC-1	50–200 nM TP, in vitro	↑CDKN1A and protein levels of phosphorylated p53, but ↓protein levels of phosphorylated c-JUN and phosphorylated NF-κB p65	[29]
Breast cancer	Human breast cancer MDA-MB-231 cells and MCF-7 cells	13–400 nM TP + 10 ng/mL TNF-α, in vitro	↓XIAP and cIAP1/2, and downstream anti-apoptotic genes activated by NF-κB	[46]
Breast cancer	MDA-MB-231 breast cancer cells	0.31–40 ng/mL TP, in vitro	↑Oxidative stress and endoplasmic reticulum stress via PERK-eIF2α pathway	[180]
Glioma	U251 cells, mouse cardiomyocytes, and LN229 cells	5–320 nM TP, in vitro	↑ROS and ↓NF-κB pathway	[47]
Burkitt lymphoma	Raji, NAMALWA, and Daudi cells	20–80 nM TP, in vitro	↑SIRT3 and↑SIRT3/GSK3β/BAX pathway	[48]
Multiple myeloma	Multiple myeloma cell line U266	40–160 nM TP, in vitro	↑G2/M cell cycle arrest and Caspase-dependent apoptosis	[49]
Multiple myeloma	Human multiple myeloma cell line U266	100 nM TP, in vitro	↓STAT3 signaling pathway	[20]
Liver cancer	HepG2 cells	10-50nM TP, in vitro	↑p53 tumor suppressor gene	[181]
Cervical cancer	Human cervical cancer cells	–	↓AKT phosphorylation and MCL-1	[50]
Pancreatic cancer and Cervical cancer	Pancreatic cancer PANC-1 and cervical adenocarcinoma HeLa cells	–	↑Caspase-3 and Caspase-8, PARP and Bid were cleaved, and↓BCL-2	[182]
Pancreatic cancer	PANC-1 and MiaPaCa-2 cells	50–200 nM TP, in vitro	↓HSP70 mRNA and protein levels	[173]
Pancreatic cancer	In situ pancreatic tumors in nude mice	0.2 mg/kg TP, i.p	↓HSP70 mRNA and protein levels	[173]
Endometrial cancer	HEC-1B cells	10–320 nM TP, in vitro	↑Caspase-3/9 and↓BCL-2 without altering BAX levels	[182]
Melanoma	SK-MEL5 and SK-MEL-28 cells, as well as HaCaT cells	10–40 nM TP, in vitro	↓SRC-ERK signaling	[183]
Melanoma	Xenograft mouse model	150 and 300 μg/kg TP, i.p	↓SRC-ERK signaling	[183]
Glioblastoma	U251 cells	50- 200 nM TP, in vitro	↓PROX1	[184]
Colorectal cancer	HT29 cell line	25–100 nM TP, in vitro	↓Nrf2 signaling	[185]
Prostate cancer	Human prostate cancer cell line PC3	12.5–50 nM TP, in vitro	↓IRF3, and ↑IFN signaling	[51]
Ovarian cancer	Ovarian cancer cell lines A2780 and A2780/CP70	–	↓HK 2n and ↑Hsa-mir-6751	[186]
Epithelial ovarian cancer	COC1/DDP cells	1–100 ng/mL TP, in vitro	↓PI3K/AKT pathway	[187]
Epithelial ovarian cancer	In situ rat model of ovarian cancer	0.025–0.1 mg/kg TP, i.p	↓PI3K/AKT pathway	[187]
Esophageal squamous cell carcinoma	KYSE150 and KYSE180 cells	4–8 nM TP, in vitro	↑MAPK/ERK pathway	[30]
Esophageal squamous cell carcinoma	KYSE30 and TE1cells	2 nM TP, in vitro	↓Glycolysis and ↑mitochondrial dysfunction	[31]
Esophageal squamous cell carcinoma	Xenograft model	0.45 mg/kg TP, i.p	↓Glycolysis and ↑mitochondrial dysfunction	[31]
Renal cell carcinoma	ACHN, A498, Caki-1, 769-P, and 786-O cell lines	TP + rTRAIL, in vitro	↑TRAIL-induced apoptosis and ↓HSP70	[52]

Other gene and protein abbreviations appearing in this table can be found in the Abbreviation List

TP: Triptolide; NSCLC: Non-small Cell lung Cancer; AML: Acute Myeloid Leukemia; CML: Chronic Myeloid Leukemia; s.c.: subcutaneous injection; i.p.: intraperitoneal injection; i.g.: intragastric administration; ROS: Reactive Oxygen Species

↑ = Activation or Upregulation

↓ = Inhibition or Downregulation.

#### Promoting apoptosis to treat inflammatory diseases

Triptolide has alleviated colitis symptoms by promoting apoptosis, offering a novel therapeutic strategy for the management of inflammatory bowel disease. Intraperitoneal injection of 0.07 mg/kg triptolide significantly upregulated suppressor of cytokine signaling 3 (SOCS3) protein expression in the lamina propria monocytes of interleukin-10 (IL-10)-deficient colitis mice, while concomitantly downregulating the anti-apoptotic genes *Bcl-2* and *Bcl-xl* by inhibiting STAT3 pathway. This mechanism was further validated in colonic explant experiments from Crohn's disease patients (treated with 20 ng/mL triptolide), confirming the therapeutic effect of triptolide through the regulation of IL-6/STAT3/SOCS3 pathway [53].

Moreover, triptolide has exhibited substantial therapeutic potential in rheumatoid arthritis models. 5–15 ng/mL triptolide to rheumatoid arthritis fibroblast-like synovial cells resulted in the downregulation of synovial cell exosome microRNA-221 (miR-221), the promotion of cartilage cell proliferation and secretory function, and the induction of apoptosis, thereby effectively treating rheumatoid arthritis [21]. The combination of triptolide with curcumin inhibited IL-17/NF-κB pathway in rats with rheumatoid arthritis and in MH7A cells, induced apoptosis and enhanced anti-inflammatory effects, providing a novel therapeutic strategy for rheumatoid arthritis [54].

#### Inhibiting apoptosis to alleviate ischemia/reperfusion injury

Triptolide has displayed anti-apoptotic activity in normal cells and has conferred robust protection against neurological ischemia/reperfusion injury. 5–80 ng/mL triptolide alleviated oxygen–glucose deprivation and tumor necrosis factor-alpha (TNF-α)-induced apoptosis in SH-SY5Y cells in a concentration-dependent manner. Intraperitoneal injection of 1.0 mg/kg of this drug ameliorated cerebral ischemia/reperfusion injury in murine models of ischemic stroke by inhibiting NF-κB and p38 MAPK activation [55]. Intraperitoneal injection of 20 μg/kg triptolide alleviated white matter damage in mice with chronic cerebral hypoperfusion by inhibiting microglial activation and the release of pro-inflammatory factors, and inhibited apoptosis in oligodendrocytes following oxygen–glucose deprivation by activating Sarcoma proto-oncogene, non-receptor tyrosine kinase (SRC)/AKT/ Glycogen Synthase Kinase-3 Beta (GSK3β) pathway, thus exerting concentration-dependent protective effects (0.001–0.1 nM) [56]. Furthermore, triptolide inhibited apoptosis by suppressing fractalkine/C-X3-C motif chemokine receptor 1 (CX3CR1) pathway in rats [57] or NF-κB/p53 upregulated modulator of apoptosis (PUMA) pathway [58], thereby alleviating brain ischemia/reperfusion injury.

Similarly, triptolide inhibited cardiac myocyte apoptosis, effectively alleviating myocardial ischemia/reperfusion injury. Mechanistically, triptolide synergistically inhibited NF-κB, ROS, and ERK1/2 pathways, induced anti-inflammatory, antioxidant, and anti-apoptotic activities, resulting in a substantial reduction in ischemia/reperfusion injury in rat hearts and H9C2 cells [59].

#### Regulating apoptosis to protect kidneys

Triptolide has demonstrated renal protective effects through differential regulating apoptosis, and its impact has varied depending on the cell type, promoting apoptosis in immune nephropathy while suppressing it in glomerulosclerosis [60, 61].

In tonsillar mononuclear cells derived from patients with Immunoglobulin A (IgA) nephropathy, 10–30 ng/mL triptolide downregulated the levels of anti-apoptotic proteins BCL-2 and BCL-XL in a dose-dependent manner, concomitantly upregulated the pro-apoptotic protein BAX, resulting in promoting apoptosis of immune cells under pathological conditions, providing a novel therapeutic option for the clinical treatment of IgA nephropathy [60].

In terms of podocyte protection, triptolide has been demonstrated to exhibit anti-apoptotic effects. 10 ng/mL triptolide downregulated the expression of apoptotic gene growth arrest and DNA-damage-inducible 45 beta b in zebrafish podocytes, blocked NF-κB/growth arrest and DNA-damage-inducible 45 beta b signaling, attenuated podocyte apoptosis, and significantly ameliorated proteinuria [61]. Furthermore, 160 mg/kg triptolide (intragastric administration) ameliorated focal segmental glomerulosclerosis in rats. Mechanistically, triptolide inhibited IL-4/STAT6 pathway, upregulated key podocyte molecules nephrin and podocin, and suppressed p53-mediated podocyte apoptosis, suggesting that it may be a potential therapeutic option for focal segmental glomerulosclerosis [62].

#### Inhibiting apoptosis to protect nervous system

Triptolide has produced neuroprotective effects through inhibiting apoptotic pathways and diminishing oxidative damage in nervous system [63]. In an amyloid-β protein fragment 25-35-induced differentiated PC12 cell model, 0.01–1 nM triptolide significantly reduced nerve cell apoptosis, with the mechanism involving upregulation of superoxide dismutase activity while inhibiting reactive oxygen species, H_2_O_2_, and malondialdehyde, thereby counteracting amyloid-β toxicity [64]. Furthermore, intraperitoneal injection of 5 μg/kg triptolide reduced oxidative stress levels in the hippocampus of rats with vascular dementia, decreased neuronal apoptosis, and improved learning and memory impairments by activating Sirtuin 1 (SIRT1)/peroxisome proliferator-activated receptor gamma coactivator 1-alpha (PGC-1α) pathway, providing a potential therapeutic strategy for neurodegenerative diseases [65].

#### Promoting apoptosis to modulate immune system

Triptolide has exhibited considerable immunosuppressive properties [66]. In aortic transplantation mice, subcutaneous injection of 0.5 mg/kg triptolide resulted in a significant alleviation of thickening of the endothelium in allograft vessels. In addition, it exhibited a dose-dependent inhibition of MOVAS-1 cell activity (5–80 ng/mL), reduced the proportion of interferon-gamma (IFN-γ)-positive T lymphocytes, decreased the secretion of IFN-γ and its inducible factors (C-X-C motif chemokine ligand 9 (CXCL9) and CXCL10, and inhibited vascular smooth muscle cell proliferation while inducing their apoptosis, offering a substantial scientific evidence for the development of novel anti-rejection strategies [67].

### Triptolide induces apoptosis leading to multi-organ toxicities

#### Induction of apoptosis leading to reproductive toxicity

Triptolide has damaged spermatogenic cells or caused structural injury to testicular tissue through inducing oxidative stress and activating the mitochondrial apoptosis pathway, ultimately impairing male reproductive function [68]. In male C57BL/6J mice, intraperitoneal administration of 0.2 mg/kg triptolide induced oxidative stress, triggered mitochondrial apoptosis in spermatogonia, and caused abnormalities in mitochondrial morphology and structure, leading to severe testicular damage. N-acetyl-L-cysteine mitigated triptolide-induced testicular damage and apoptosis by alleviating oxidative stress [69]. Mechanistically, triptolide suppressed SIRT1/nuclear factor erythroid 2-related factor 2 (Nrf2) pathway, triggered oxidative stress, and altered mitochondrial morphology and membrane potential, disrupting rat testicular structure (100 μg/kg, intraperitoneal injection) and inducing apoptosis in TM4 cells (40–640 nM); melatonin alleviated triptolide-induced damage to testicular supporting cells by modulating the crosstalk between SIRT1 and Nrf2 [70].

In rats and the Leydig cell line TM3, triptolide induced the expression of dynamin-related protein 1 (Drp1), disrupted mitochondrial dynamic stability, and activated the mitochondrial apoptosis pathway, ultimately inducing testicular interstitial cell apoptosis in vitro and in vivo, exhibiting significant reproductive toxicity. The selective Drp1 inhibitor Mdivi-1 attenuated reproductive toxicity of triptolide [23].

#### Induction of apoptosis leading to hepatotoxicity

The hepatotoxicity of triptolide has represented a significant barrier to its clinical application [71, 72]. The underlying mechanism of this process involved endoplasmic reticulum stress-mediated endogenous apoptosis [22], extrinsic apoptosis through the activation of death receptor pathways [73], and a synergistic effect of these two pathways [74].

Intragastric administration of 500 μg/kg triptolide to female C57BL/6J mice induced ROS generation and activated glucose-regulated protein 78 (GRP78)-activating transcription factor 4 (ATF4)-C/EBP homologous protein (CHOP) axis through proteasome inhibition, which in turn enhanced endoplasmic reticulum stress-related apoptosis and caused hepatocyte damage. The ROS inhibitor N-acetylcysteine alleviated triptolide-induced hepatotoxicity by reducing the expression of endoplasmic reticulum stress-related apoptotic proteins and ROS levels [22].

In C57BL/6J mice, intragastric administration of 500 μg/kg triptolide for 7 consecutive days inhibited X-linked inhibitor of apoptosis protein (XIAP) expression, enhanced hepatocyte sensitivity to Fas cell surface death receptor (Fas)/Fas ligand (FasL) pathway, and induced hepatocyte apoptosis mediated via death receptor pathway. Additionally, treatment of AML12 cells with 25–50 nM triptolide upregulated miR-137, inhibited XIAP protein expression, and rendered hepatocytes more susceptible to apoptosis, ultimately leading to hepatotoxicity [73].

In HepaRG cells, 100–400 nM triptolide elevated ROS levels, dissipated mitochondrial membrane potential, and shifted the apoptotic balance through upregulation of Fas, BAX, p53 and cleaved Caspase-3/8/9, coupled with BCL-2 downregulation, indicating that triptolide inhibited HepaRG cell proliferation and induced apoptosis through Fas death pathway and mitochondrial pathway, ultimately causing hepatotoxicity [74].

#### Induction of apoptosis leading to nephrotoxicity

Triptolide has been shown to exhibit potent anti-renal cell carcinoma activity by sensitizing Renca cells to tumor necrosis factor-related apoptosis-inducing ligand (TRAIL)-induced apoptosis and suppressing heat shock protein 70 (HSP70) expression [52]. However, it has simultaneously triggered apoptosis in renal cells by regulating oxidative stress-related pathways, exacerbating kidney injury [75].

Triptolide exhibited significant cytotoxicity towards renal tubular epithelial cell line NRK-52E, specifically manifested as 160 nM triptolide inhibited the nuclear translocation of the transcription factor Nrf2 through the activation of GSK3β and the promotion of FYN proto-oncogene, Src family tyrosine kinase (Fyn) protein nuclear translocation, resulting in oxidative stress and renal tubular epithelial cell damage and ultimately contributing to nephrotoxicity [24]. In addition, triptolide increased ROS, lactate dehydrogenase, malondialdehyde (MDA) and glutathione (GSH) while reducing superoxide dismutase, inducing apoptosis in HK2 cells and renal cells of BALB/c mice, resulting in renal impairment. Triptriolide counteracted triptolide-induced apoptosis in renal cells by suppressing oxidative stress [76].

#### Induction of apoptosis leading to cardiotoxicity

Triptolide has induced cardiotoxicity by triggering myocardial cell apoptosis through mitochondrial damage via the activation of p53-BAX axis or the suppression of Nrf2/Kelch-like ECH-associated protein 1 (Keap1)-nuclear respiratory factor 1 (NRF1) axis. Treating H9c2 cells and primary myocardial cells with 160 nM triptolide for 24 h significantly upregulated the expression of p53 protein and its downstream target genes, leading to increased mitochondrial outer membrane permeability and mitochondrial dysfunction. In p53 knockout mice, an intravenous injection of 1.2 mg/kg triptolide did not cause significant cardiac tissue damage, and both p53 and BAX antagonists mitigated triptolide-induced cardiotoxicity, further confirming that triptolide exerted cardiotoxicity through the activation of p53-BAX axis [77].

Furthermore, triptolide induced cardiotoxicity by enhancing Nrf2-Keap1 interaction and disrupting Nrf2-PGC-1α binding which promoted Nrf2 ubiquitination and degradation [78], while simultaneously suppressing NRF1 expression, collectively leading to oxidative stress, mitochondrial dysfunction, and ultimately cardiomyocyte apoptosis; calycosin coordinately activated both Nrfr2 and NRF1 pathways, representing a promising strategy to mitigated triptolide-induced cardiotoxicity [79].

## Bidirectional autophagy modulation by triptolide generates pharmacological effects and toxicities

Triptolide, a potential bidirectional autophagy regulator, has been shown to exhibit dose-dependent effects. At low doses, it has activated survival autophagy through pathways such as ROS/c-Jun N-terminal kinase (JNK), demonstrating pleiotropic pharmacological benefits. In contrast, high doses have induced autophagic cell death, which has been associated with toxic side effects [80].

**Fig. 3:**
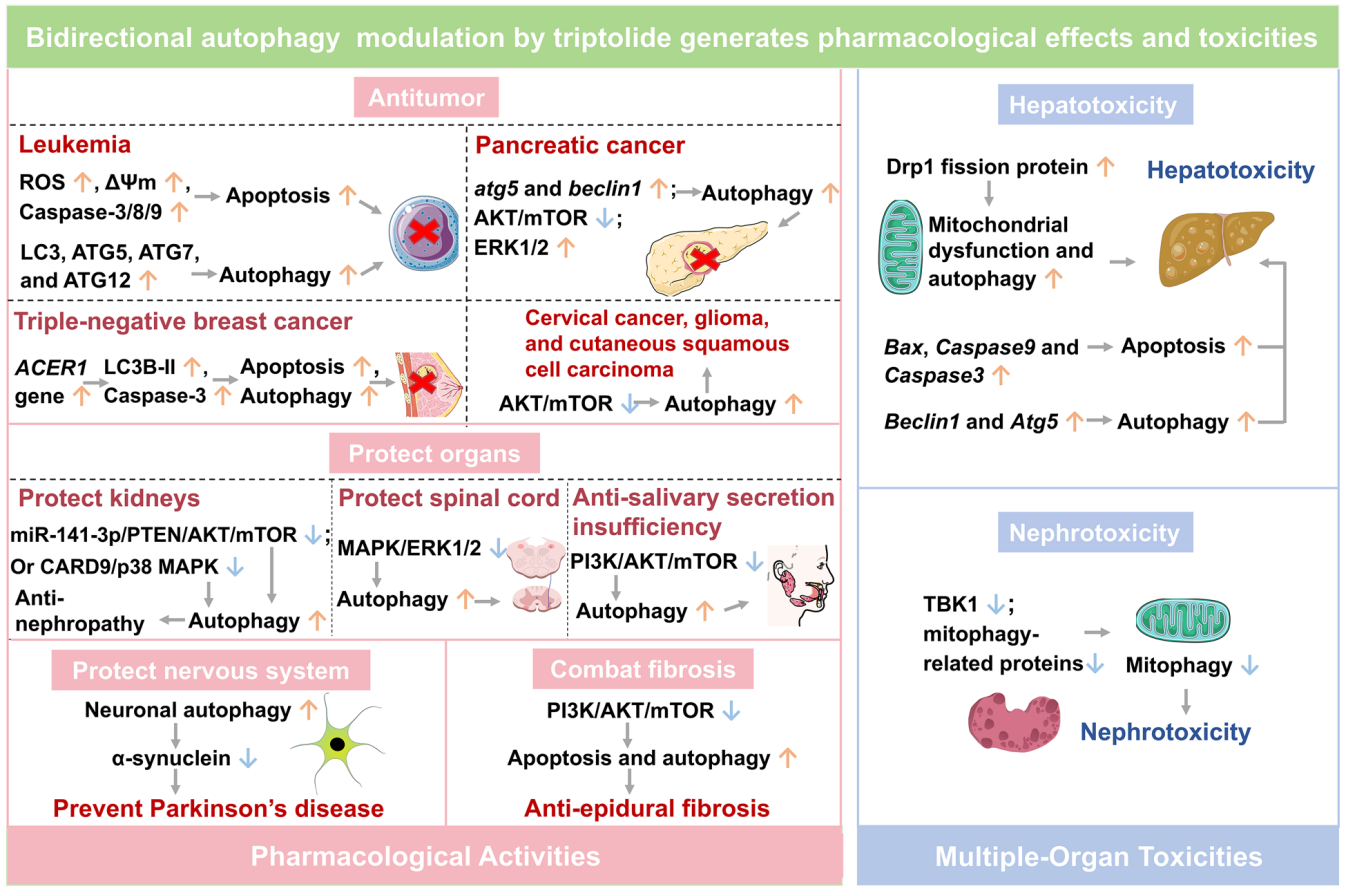
Bidirectional autophagy modulation by triptolide generates pharmacological effects and toxicities. ↑: Activation or Upregulation; ↓: Inhibition or Downregulation. Other gene and protein abbreviations appearing in this figure can be found in the Abbreviation List

Triptolide has exhibited potent anti-tumor activity by finely modulating the cell death pathway network, inducing synergistic crosstalk between autophagy and apoptosis, with demonstrated efficacy against pancreatic cancer, leukemia, and triple-negative breast cancer [81–83]. Regarding organ protection, triptolide has restored autophagy function through multi-pathway regulation, mitigating diabetic nephropathy, IgA nephropathy, spinal cord injury, and salivary gland dysfunction [84–87]. Its neuroprotective effects have arisen from enhanced neuronal autophagy, promoting the clearance of pathogenic α-synuclein aggregates, suggesting therapeutic potential in Parkinson’s disease [88]. Additionally, triptolide has exerted anti-fibrotic effects by inhibiting PI3K/AKT/mTOR pathway, thereby attenuating renal and surgery-induced epidural fibrosis [86, 89]. However, its therapeutic potential has been restricted by hepatotoxicity and nephrotoxicity, primarily driven by mitochondrial dysfunction and dysregulated autophagy pathways [90, 91] (Fig. 3).

### Autophagic modulation by triptolide in pharmacological activities

#### Promoting autophagy to exert antitumor effects

Triptolide has regulated the autophagy pathway through multiple molecular mechanisms and has exhibited significant antitumor activity in cancer. Triptolide (20–120 nM) induced ROS accumulation, increased mitochondrial membrane potential, and activated Caspase-8, -9, and -3 in WEHI-3 cells. It also promoted autophagy by enhancing microtubule-associated protein 1 light chain 3 (LC3), autophagy related 5 (ATG5), ATG7, and ATG12 expression, suggesting that triptolide synergistically activated both apoptosis and autophagy pathways to exert anti-leukemic effects [92].

In pancreatic cancer, triptolide activated autophagy by downregulating PUM1 and enhancing tumor cell sensitivity to TRAIL [93]. It also induced both caspase-dependent and caspase-independent autophagic cell death in various cell lines, and this autophagy induction depended on the *atg5* and *beclin1* genes and was accompanied by the inhibition of AKT/mTOR pathway and the activation of ERK1/2 pathway, indicating that triptolide was an effective chemotherapeutic agent for treating pancreatic cancer [82].

In triple-negative breast cancer, 25 nM triptolide induced both autophagy and apoptosis in MDA-MB-231 cells by downregulating p62, upregulating the LC3B-II and activating Caspase-3 making it a potential anticancer lead compound for triple-negative breast cancer [94]. Further research revealed that alkaline ceramidase 1 (*ACER1*) gene overexpression enhanced triptolide-induced apoptosis in MDA-MB-231 cells, promoted autophagosome formation (LC3B-II upregulation and p62 downregulation) and inhibited cell migration and invasion, establishing *ACER1* as a potential target for triptolide intervention in triple-negative breast cancer [95].

In addition, triptolide inhibited AKT/mTOR signaling and induced autophagy and apoptosis in cancer cells, exerting cytotoxicity in cervical cancer [96], glioma [83], and cutaneous squamous cell carcinoma [97], collectively highlighting its broad-spectrum antitumor properties.

#### Promoting autophagy to protect organs

Triptolide has restored cellular autophagy capacity and has exerted significant renal protective effects against various diseases. For instance, triptolide downregulated miR-141-3p expression, and upregulated phosphatase and tensin homolog deleted on chromosome 10 (PTEN) levels, then inhibited AKT/mTOR pathway to restore cellular autophagy and alleviate diabetic fibrosis [98]. Moreover, triptolide promoted autophagy by downregulating caspase recruitment domain-containing protein 9 (CARD9)/p38 MAPK pathway, which inhibited mesangial cell proliferation in both IgA nephropathy mice (300 μg/kg, intragastric administration) and IgA1-induced human mesangial cells (20 ng/mL), providing a novel therapeutic approach for IgA nephropathy [85].

In a spinal cord injury model, intraperitoneal injection of 0.2 mg/kg triptolide induced autophagy by inhibiting MAPK/ERK1/2 pathway, exerting a protective effect and reducing kidney damage [84].

Furthermore, 2.5 ng/mL triptolide promoted autophagy and M2 polarization in aged macrophages by inhibiting PI3K/AKT/mTOR pathway, and improved salivary gland structure and function, which potentially prevented age-related salivary secretion insufficiency [87].

#### Promoting autophagy to protect nervous system

Triptolide has been shown to effectively promote the clearance of α-synuclein by neuronal autophagy, facilitating the removal of damaged or senescent neurons [99]. 0.5–50 nM triptolide reduced the levels of α-synuclein in neuronal cells and primary cortical neurons via the activation of autophagy, underscoring its therapeutic potential for Parkinson’s disease and other neurodegenerative disorders characterized by protein aggregation [88].

#### Promoting autophagy to combat organ fibrosis

In addition to mitigating diabetic renal fibrosis by restoring autophagy via miR-141-3p/PTEN/AKT/mTOR pathway [98], triptolide has also demonstrated significant efficacy against surgery-related epidural fibrosis. 2–8 μg/mL triptolide significantly suppressed human fibroblast proliferation by inhibiting PI3K/AKT/mTOR pathway, while simultaneously promoting apoptosis and autophagy. In a rat model, local administration of 0.2 and 0.4 mg/mL triptolide effectively alleviated postoperative fibrosis, offering a potential therapeutic strategy for preventing surgery-related epidural fibrosis [89].

### Triptolide has induced autophagy leading to multi-organ toxicities

#### Induction of autophagy leading to hepatotoxicity

Triptolide has induced hepatotoxicity by causing mitochondrial dysfunction and the activation of mitochondrial autophagy. For instance, triptolide increased the expression of Drp1 fission protein in LO2 cells, along with the colocalization of autophagosomes with mitochondria; the selective Drp1 inhibitor Mdivi-1 alleviated triptolide-induced hepatotoxicity. In rat liver tissue treated with 400 μg/kg triptolide, mitochondrial fission was significantly activated, further indicating that targeting the mitochondrial fission and autophagy pathways represented a new therapeutic approach against triptolide-induced hepatotoxicity [90].

Triptolide has also induced hepatotoxicity by activating the pathways of autophagy and apoptosis [100]. In a zebrafish model, exposure to 0.2–0.8 μM triptolide activated Fas-Caspase-8 apoptosis pathway, characterized by the upregulation of the pro-apoptotic genes *Bax*, *Caspase-9* and *Caspase-3*. Meanwhile, the expression of the autophagy-related genes *Beclin1*, *Atg5*, *Atg3* and *Lc3* was significantly increased, with the most prominent changes observed in *Beclin1* and *Atg5*, suggesting that death receptor *Fas* may be the primary target of triptolide-induced hepatotoxicity [101].

#### Inhibition of autophagy leading to nephrotoxicity

The mechanism of triptolide-induced nephrotoxicity has been linked to its disruption of mitochondrial function and suppression of mitophagy. In Sprague–Dawley rats, intragastric administration of 0.5–2.0 mg/kg triptolide downregulated TANK-binding kinase 1 (TBK1) and mitophagy-related proteins, reducing mitochondrial autophagosome formation and impairing renal function; TBK1 overexpression restored mitophagy and mitochondrial function, indicating that targeting the TBK1-mitophagy pathway could be a potential therapeutic strategy to mitigate triptolide-induced nephrotoxicity [91].

## Bidirectional pyroptosis modulation by triptolide generates pharmacological effects and toxicities

Pyroptosis, also known as inflammatory necrosis, is primarily caused by the activation of inflammasomes, which activate Caspase-1 or Caspase-4/5, ultimately leading to cell membrane rupture, release of cellular contents, and a strong inflammatory response [102].

**Fig. 4:**
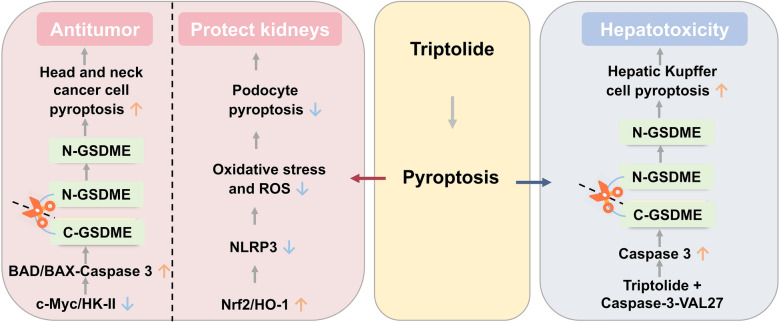
Bidirectional pyroptosis modulation by triptolide generates pharmacological effects and toxicities. ↑: Activation or Upregulation; ↓: Inhibition or Downregulation. Other gene and protein abbreviations appearing in this figure can be found in the Abbreviation List

Triptolide has exhibited various pharmacological and toxic effects through pyroptosis regulation. In antitumor research, this compound has been shown to enhance immune responses and inhibit cancer cell growth via the activation of gasdermin E (GSDME)-mediated pyroptosis pathway [103]. For renal injury, triptolide has demonstrated the renal protection to alleviate podocyte damage through Nrf2/ROS/NOD-like receptor family, pyrin domain-containing 3 (NLRP3) axis [104]. However, triptolide has also been reported to induce Kupffer cell pyroptosis by activating the Caspase-3/GSDME pathway, resulting in hepatotoxicity [105] (Fig. 4).

### Pyroptotic modulation by triptolide in pharmacological activities

#### Promoting pyroptosis to exert anti-tumor effects

Triptolide has demonstrated both antitumor immunity enhancement and cytotoxicity towards head and neck cancer cells through agitation of GSDME-mediated pyroptosis. Treating HK1, FaDu, and C666-1 cells with 50 nM triptolide significantly suppressed cellular myelocytomatosis viral oncogene homolog (c-Myc) and mitochondrial hexokinase II expression, activated the Bcl-2-associated death promoter (BAD)/BAX-Caspase 3 cascade, subsequently induced Caspase 3-mediated cleavage of GSDME, and triggered tumor cell pyroptosis, establishing a new paradigm for triptolide as a pyroptosis-inducing agent in cancer therapy [103].

#### Inhibiting pyroptosis to protect the kidneys

Triptolide has been shown to alleviate podocyte damage in diabetic nephropathy by regulating Nrf2/ROS/NLRP3 axis. Specifically, intragastric administration of triptolide (100 μg/kg/day) activated Nrf2/heme oxygenase 1 (HO-1) pathway while inhibiting NLRP3 inflammasome pathway, reduced oxidative stress and ROS levels, attenuated pyroptosis, and improved renal function and histopathological damage in diabetic nephropathy mice [104].

### Triptolide induces pyroptosis leading to hepatotoxicity

Twenty–200 μM triptolide bound to the VAL27 site of hepatic Kupffer cells, induced the cleavage of GSDME to generate N-GSDME by promoting Caspase-3 maturation and activation, and ultimately triggered hepatic cell pyroptosis. Knocking out Caspase-3 or administration of Caspase-3 inhibitors counteracted triptolide-induced liver damage in mice, which further confirmed that triptolide exerted hepatotoxicity through the activation of GSDME/CASP3 axis [105].

## Bidirectional ferroptosis modulation by triptolide generates pharmacological effects and toxicities

**Fig. 5:**
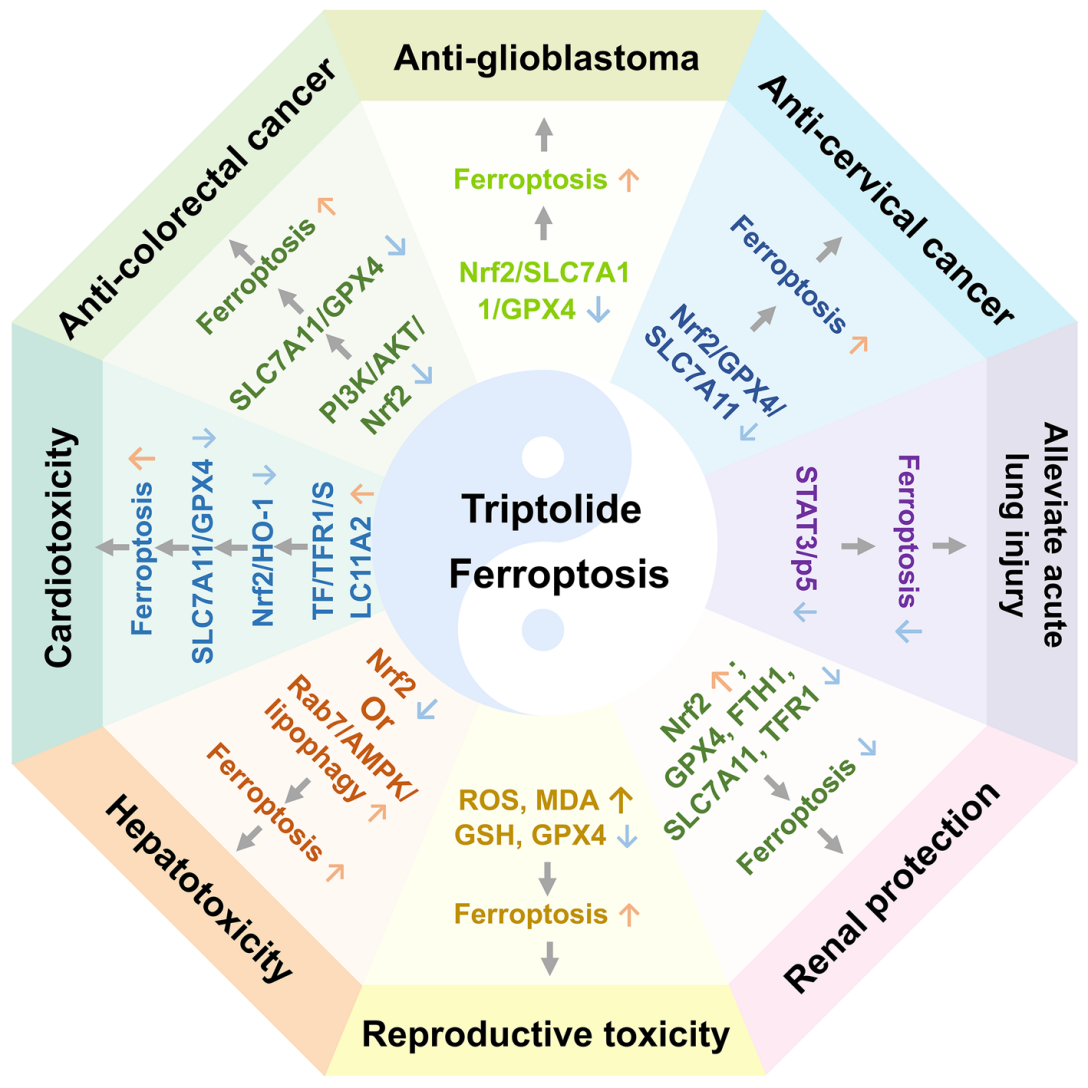
Bidirectional ferroptosis modulation by triptolide generates pharmacological effects and toxicities. ↑: Activation or Upregulation; ↓: Inhibition or Downregulation. Other gene and protein abbreviations appearing in this figure can be found in the Abbreviation List

Ferroptosis is an iron-dependent cell death driven by the accumulation of lethal lipid peroxides [106]. Extensive investigations have characterized triptolide as a potent inducer of ferroptosis in various cancer cell lines, and a tumor suppressor or a drug sensitizer in standard chemotherapy regimens [107–110]. Regarding organ protection, triptolide has significantly suppressed ferroptosis in lung and kidney tissues, exerting protective effects against acute lung injury and diabetic nephropathy [111, 112]. Nonetheless, triptolide has also been associated with ferroptosis-related toxicities, contributing to reproductive, cardiac, and hepatic damage [113–115] (Fig. 5).

### Ferroptotic modulation by triptolide in pharmacological activities

#### Promoting ferroptosis to exert anti-tumor effects

Triptolide has induced ferroptosis in tumor cells by regulating Nrf2 and its downstream targets [glutathione peroxidase 4 (GPX4), solute carrier family 7 member 11 (SLC7A11)], exerting broad-spectrum antitumor effects [107, 108, 116]. 25–100 nM triptolide downregulated SLC7A11 and GPX4 expression via the inhibition of PI3K/AKT/Nrf2 pathway, promoting ferroptosis and producing cytotoxicity in colorectal cancer cells (HCT116 and LoVo). In transplanted tumor, intraperitoneal injection of triptolide (5 mg/kg) activated ferroptosis by suppressing SLC7A11/GPX4 axis and inhibited tumor growth in colorectal cancer [107]. Furthermore, triptolide-loaded hydrogels activated ferroptosis in glioblastoma cells by the suppression of Nrf2/SLC7A11/GPX4 pathway, significantly prolonging survival in mice with transplanted recurrent glioblastoma [108].

Triptolide (10–50 nM) promoted peroxidized lipid accumulation by inhibiting Nrf2/GPX4/SLC7A11 axis, then induced ferroptosis in HeLa and SiHa cervical cancer cells; and 10–20 mg/kg triptolide significantly inhibited cervical cancer xenograft growth, supporting its potential as a therapeutic candidate for cervical cancer treatment [109]. Interestingly, 40 nM triptolide reversed doxorubicin resistance in leukaemia cells through Nrf2 suppression and restored chemotherapy sensitivity, indicating that triptolide-doxorubicin combination therapy improved leukemia treatment efficacy [110].

#### Inhibiting ferroptosis to protect organs

Triptolide could inhibit ferroptosis mediated by STAT3/p53 pathway and reduce inflammation, alleviating acute lung injury. Intraperitoneal administration of triptolide (50 μg/kg/d, 7 days) significantly decreased STAT3 and p53 phosphorylation levels in lung tissue. Mechanistically, triptolide (1–1000 nM) suppressed lipopolysaccharide-induced ferroptosis in human bronchial epithelial cells (BEAS-2B) through inhibition of STAT3/p53 pathway, suggesting its therapeutic potential for acute lung injury [111].

Triptolide has also demonstrated significant renal protection in diabetic nephropathy by inhibiting ferroptosis-mediated oxidative stress. In db/db mice, triptolide significantly upregulated Nrf2 expression while downregulating GPX4, ferritin heavy chain 1 (FTH1), and SLC7A11 levels in renal tissue, suppressed transferrin receptor 1 (TFR1) production, and effectively inhibited the ferroptosis cascade. These effects collectively stabilized the glomerular podocyte cytoskeleton, reduced oxidative stress, and improved mitochondrial dysfunction, consequently ameliorating diabetic nephropathy-associated proteinuria [112].

### Triptolide induces ferroptosis leading to multi-organ toxicities

#### Induction of ferroptosis leading to reproductive toxicity

Through induction of ferroptosis in spermatogonia, triptolide has caused testicular damage and has impaired spermatogenic function. In a mouse model, triptolide significantly increased ROS and MDA levels while reducing GSH content and GPX4 expression. Mechanistically, triptolide triggered Lysine 63 (K63)-linked polyubiquitination-mediated GPX4 degradation, activated ferroptosis pathway and consequently induced testicular tissue damage and spermatogenic abnormalities (reduced sperm concentration and morphological defects), while this male reproductive toxicity was attenuated by the ferroptosis inhibitor ferrostatin-1 [113].

#### Induction of ferroptosis leading to hepatotoxicity

Triptolide has demonstrated bidirectional regulation of Nrf2 pathway, producing opposite biological effects in different tissues. In renal tissue, triptolide attenuated ferroptosis by upregulating Nrf2, thereby maintaining renal tight junction integrity in diabetic nephropathy [112]. Conversely, hepatic exposure to triptolide promoted Nrf2 degradation, triggering ferroptosis-mediated liver injury. 70 nM triptolide inhibited Nrf2 signaling in LO2 and AML12 hepatocytes, potentiating ferroptosis. Nrf2-knockout mice administered 1.2 mg/kg triptolide via intraperitoneal injection exhibited exacerbated lipid metabolic disorders and hepatotoxicity, which were ameliorated by exogenous Nrf2 overexpression [114].

Triptolide has activated RAS-associated protein RAB-7 (Rab7)-mediated lipophagy and induced hepatocyte ferroptosis, resulting in hepatotoxicity. 25–100 nM triptolide upregulated Rab7 expression by activating AMPK pathway, promoting lipid droplet degradation and free fatty acid release, which subsequently induced mitochondrial dysfunction and oxidative stress, leading to ferroptosis in the human normal liver cell line HL7702; intraperitoneal injection of 0.4–0.8 mg/kg triptolide in male C57BL/6J mice impaired liver function, activated lipophagy, and elevated ferroptosis markers, while the inhibition of lipophagy effectively alleviated triptolide-induced ferroptosis and hepatotoxicity, further confirming that triptolide triggered ferroptosis through Rab7/AMPK/lipophagy axis [117].

#### Induction of ferroptosis leading to cardiotoxicity

Through modulation of SLC7A11/GPX4 axis, triptolide has exhibited dual pharmacological effects: inducing ferroptosis to exert both antitumor activity [107, 108] and organ protection [112], while paradoxically mediating potential cardiotoxicity via the same pathway. 20–640 nM triptolide inhibited Nrf2/HO-1 antioxidant pathway by upregulating transferrin (TF)/TFR1/SLC11A2 axis, leading to iron overload, Fenton reaction-mediated ROS generation, and the accumulation of lethal lipid peroxides. Additionally, triptolide directly bound to SLC7A11, subsequently suppressed SLC7A11/GPX4 axis and induced ferroptosis in the human cardiomyocyte line AC16, ultimately causing cardiac damage. The ferroptosis inhibitor Ferrostatin-1 alleviated the cardiotoxicity, further confirming triptolide-mediated ferroptosis cardiotoxicity [115].

## Triptolide triggers cuproptosis to exert pharmacological activities

Cuproptosis is a copper-dependent cell death pathway initiated by the binding of copper to lipoylated tricarboxylic acid cycle proteins, resulting in protein aggregation, proteotoxic stress, and cell death [118]. Triptolide has induced cuproptosis in cervical cancer cells through XIAP/copper metabolism domain containing 1 (COMMD1)/ATPase copper transporting alpha (ATP7A) and beta (ATP7B)-mediated copper dysregulation, holding great promise for anticancer therapy. Targeting XIAP and modulating COMMD1, triptolide (20–160 nM) induced cuproptosis in HeLa and SiHa cells, characterized by downregulated expression of copper export proteins ATP7A/B but unaltered expression of copper import protein copper transporter 1. Moreover, administration of 0.2–0.6 mg/kg triptolide markedly suppressed tumor growth in nude mice bearing cervical cancer xenografts, supporting the therapeutic potential of triptolide-induced cuproptosis in cervical cancer treatment [119].

## Triptolide triggers necroptosis leading to hepatotoxicity

Necroptosis, a form of PCD mediated by receptor-interacting protein kinase 1 (RIPK1) and RIPK3 kinases, is triggered by pro-inflammatory extracellular stimuli and involves necrosome formation and mixed lineage kinase domain-like (MLKL) activation, representing a highly relevant process in liver pathophysiology [120, 121].

Triptolide induced hepatic hypersensitivity, leading to hepatocyte apoptosis and necroptosis. Mechanistically, triptolide inhibited NF-κB-dependent transcriptional activity and suppressed FLICE-like inhibitory protein (FLIP) expression, sensitizing mice and human hepatic cell line LO2 to lipopolysaccharide and TNF-α, which subsequently triggered hepatocyte apoptosis and necroptosis. Etanercept (a TNF-α inhibitor) or exogenous FLIP overexpression effectively blocked necroptosis-related proteins, including RIPK1, MLKL, and P-MLKL, and markedly reduced triptolide-induced hepatotoxicity, underscoring the pivotal role of NF-κB/FLIP axis in mediating triptolide-associated hepatotoxicity [16]. While necroptosis has been implicated in triptolide-induced hepatotoxicity in the studied model [16], whether it contributes to toxicity in other organs remains to be fully elucidated.

## Triptolide triggers PANoptosis leading to multi-organ toxicities

Currently, only the hepatotoxicity and nephrotoxicity of triptolide induced by PANoptosis have been experimentally confirmed. Whether PANoptosis elicits other pharmacological or toxic effects of triptolide in different tissues or under other pathological conditions remains to be investigated. Triptolide has been shown to simultaneously activate pyroptosis, apoptosis, and necroptosis, forming PANoptosome complexes and inducing PANoptosis [122], ultimately leading to hepatotoxicity and nephrotoxicity in vivo and in vitro. Mechanistically, in mouse macrophages (J774A.1) and human primary macrophages, 6.25–200 nM triptolide triggered ASC speck formation (pyroptosis induction), Caspase-8 activation (apoptosis induction), and RIPK3 aggregation (necroptosis induction), while promoting colocalization of ASC with RIPK3 or Caspase-8. In C57BL/6J mice, intraperitoneal injection of 1–2 mg/kg triptolide induced cleaved CASP3 (apoptosis), GSDME-NT, CASP1p10, GSDMD-NT (pyroptosis), and p-MLKL (necroptosis) in liver tissue, confirming PANoptosis-mediated organ toxicities and suggesting its inhibition as a potential therapeutic strategy [123].

Based on the PCD regulatory characteristics and multi-organ toxicities limitations of triptolide (Table 2), nano-delivery systems have become a potential strategy to break through the bottleneck of clinical translation.

**Table 2:** Programmed cell death-mediated multi-organ toxicities induced by triptolide

Toxicities	Cell lines/models	Dosage	Molecular mechanisms	Refs.
Hepatotoxicity	C57BL/6 mice	500 μg/kg TP, i.g	↓XIAP protein, ↑the sensitivity of hepatocytes to Fas/FasL pathway, ↑apoptosis	[73]
	AML12 cell line	25 and 50 nM TP, in vitro	↓XIAP protein, ↑the sensitivity of hepatocytes to Fas/FasL pathway, ↑apoptosis	[73]
	HepaRG cells	100–400 nM TP, in vitro	↑Fas death pathway and mitochondrial apoptosis	[74]
	C57BL/6J female mice	500 μg/kg TP, i.g	↓Proteasome and ↑ROS production via ATF4, thereby ↑ERS-related apoptosis	[22]
	LO2 cells	–	↑Drp1-related mitochondrial dysfunction and mitochondrial autophagy	[90]
	Zebrafish	0.2–0.8μM TP, *waterborne administration*	↑Autophagy-related genes *Beclin1*,*Atg5*,*Atg3* and *Lc3*	[101]
	Zebrafish	300 nM TP, *waterborne administration*	↑Oxidative stress, lipid metabolism, autophagy, and apoptosis	[100]
	Kupffer cells	20–200 μM TP, in vitro	↑Caspase-3-GSDME pyroptosis	[105]
	C57BL/6 mice and Caspase-3 knockout mice	1000 μg/kg TP, i.g	↑Caspase-3-GSDME pyroptosis	[105]
	LO2 cells and AML12 cells	70 nM TP, in vitro	↓Nrf2 pathway, ↑ferroptosis	[114]
	Nrf2—/—mice and C57BL/6J mice	1.2 mg/kg TP, i.p	↓Nrf2 pathway, ↑ferroptosis	[114]
	Male Wistar rats	2 mg/kg TP, i.p	↑Proapoptotic proteins cytochrome C, cleaved Caspase-3 and BCL-2-associated X	[188]
	Female C57BL/6J mice	0.1 mg/kg TP, i.p	↑Hepatocyte apoptosis via mitochondrial dysfunction	[189]
	AML12 cells	50 nM TP, in vitro	↑JNK pathway, ↓hepatocyte apoptosis	[189]
	LO2 cells	50 nM TP, in vitro	↑Hepatotoxicity-associated proteins PKCα and FIS1, ↑mitochondrial apoptosis	[190]
	Female BALB/c mice	500 μg/kg TP, i.p	↑Hepatotoxicity-associated proteins PKCα and FIS1, ↑mitochondrial apoptosis	[190]
	Human normal liver cell line HL7702	25–100 nM TP, in vitro	↑Rab7-mediated lipophagy, ↑hepatic cells ferroptosis	[117]
	Male C57BL/6J mice	0.4–0.8 mg/kg TP, i.p	↑Rab7-mediated lipophagy, ↑hepatic cells ferroptosis	[117]
	Mice	500 μg/kg TP, i.g. + 0.1 mg/kg LPS, i.p	↓NF-κB and FLIP, ↑hepatocytes apoptosis and necroptosis	[16]
	Human hepatic cell line LO2	25 nM TP + 50 ng/mL TNF-α, in vitro	↓NF-κB and FLIP, ↑hepatocytes apoptosis and necroptosis	[16]
Nephrotoxicity	NRK-52E cells	80–320 nM TP, in vitro	↑Apoptosis by promoting Nrf2 degradation through ubiquitination via GSK3β/Fyn pathway	[24]
	HK2 cell	5–640 nM TP, in vitro	↑Oxidative stress, ↑nephrocyte apoptosis	[76]
	BALB/c mice	1 mg/kg TP, i.g	↑Oxidative stress and ↑renal function parameters, ↑renal injury	[76]
	Sprague–Dawley rats	0.5–2.0 mg/kg/d TP, i.g	↓TBK1 and mitophagy-related proteins	[91]
Hepatotoxicity and Nephrotoxicity	J774A.1 mouse macrophage cell line and primary macrophages	6.25-200nM TP, in vitro	PANoptosome assembly	[123]
	C57BL/6J mice	1 and 2 mg/kg TP, i.p	CASP3, GSDME-NT, and p-MLKL cleavage, PANoptosome assembly	[123]
Cardiotoxicity	H9c2 cells and rat primary cardiomyocytes	160 nM TP, in vitro	↑BAX-induced mitochondrial-mediated apoptosis	[77]
	p53 − / − mice and C57BL6/J mice	1.2 mg/kg TP, i.v	↑BAX-induced mitochondrial-mediated apoptosis	[77]
	Male Balb/c mice	1.5 mg/kg TP, i.p	↓Nrf2/Keap1-NRF1 axis, ↑cardiomyocyte apoptosis	[79]
	H9C2 cardiomyocytes	200 nM TP, in vitro	↓Nrf2/Keap1-NRF1 axis, ↑cardiomyocyte apoptosis	[79]
	AC16 Human cardiac muscle cell line	20–640 nM TP, in vitro	↑SLC7A11/GPX4 inactivation-mediated ferroptosis	[115]
Reproductive toxicity	C57BL/6J male mice	0.2 mg/kg TP, i.p	↑Mitochondrial apoptosis pathway	[69]
	Sprague–Dawley male rats	100 μg/kg, i.p	↓SIRT1/Nrf2 pathway, ↑apoptosis	[70]
	TM4 cells	40–640 nM, in vitro	↓SIRT1/Nrf2 pathway, ↑apoptosis	[70]
	Adult male Sprague–Dawley rats	400 μg/kg TP, i.g	↑Drp1, ↑mitochondrial apoptosis pathway	[23]
	Testicular interstitial cell line TM3	–	↑Leydig cell apoptosis by disrupting mitochondrial dynamics in rats	[23]
	Male mice	100 μg/kg TP, i.p	↑K63-linked polyubiquitination of GPX4 in spermatogonia, ↑ferroptosis	[113]
	GC-2spd cell line	0.2 μM TP, in vitro	↑K63-linked polyubiquitination of GPX4 in spermatogonia, ↑ferroptosis	[113]

Other gene and protein abbreviations appearing in this figure can be found in the Abbreviation List

TP: Triptolide; i.v.: intravenous; i.p.: intraperitoneal injection; i.g.: intragastric administration; Drp1: Dynamin-related protein 1; LPS: Lipopolysaccharide

↑ = Activation or Upregulation

↓ = Inhibition or Downregulation

## Nanostrategies for attenuating organ toxicity and potentiating therapeutic effects of triptolide

By enhancing targeted delivery to disease sites [124, 125], promoting cellular uptake [126, 127], and selective accumulation in pathological tissues [128, 129], nanotechnology-based approaches have effectively reduced multi-organ toxicities of triptolide. Furthermore, nano-delivery systems have significantly enhanced the therapeutic efficacy of triptolide through combination therapy [130, 131], microenvironment-responsive release [132, 133], and optimized pharmacokinetic behavior [134–137], offering a promising pathway toward its clinical application (Table 2).

However, all current triptolide nano-delivery formulations remain at the preclinical research stage, which fully exposes the severity of its industrialization prospects. Specifically, (1) Existing formulations have generally suffered from low drug loading (TPLP-HA has exhibited only 2.17% [127] and TP-FPNPs only 1.11% [126]) and burst release phenomena (DT/Pep1 has released explosively at pH 7.4 [138], and TPL-NPs has shown sudden release after 12 h [139]). (2) Numerous intelligent formulations have performed well in controlled laboratory environments, factors such as temperature gradients, pH fluctuations, and uneven illumination in industrial-scale production reactors have led to the loss of predictability of their response behavior [132, 140]. (3) The long-term safety and in vivo metabolic characteristics of TP-TPBC-PEG as well as the long-term stability of TF-TP@LIP all require costly and time-consuming research verification [124, 141] (Table 3).

**Table 3:** Summary of disease applications, advantages, and limitations of triptolide nanoformulations

Nanoformulations	Diseases	Advantages	Limitations	Refs.
TPL-HSP	Bladder cancer	TPL-HSP outperformed the free TP by suppressing tumor volume to 0.173 cm^3^ in mice	Release rate required further optimization	[156]
TPLP-HA	Breast cancer	Increased LD_50_ by 2.36-fold (to 3.07 mg/kg) vs. free TP (1.30 mg/kg), achieved 51.6% tumor inhibition, and alleviated hepatorenal toxicity	Relatively low drug loading capacity (only 2.17%)	[127]
DT/Pep1	Breast cancer	DT/Pep1 achieved a high tumor suppression rate of 68.4%	Drug burst release occured at pH 7.4	[138]
TPL@nano-gel	Breast cancer	TPL@nino-gel significantly prolonged median survival in tumor-bearing mice to > 36 days (vs. 22 days with free TP), without significant weight loss	Drug loading efficiency = 4.52%	[149]
Cel + TP/RBCm@R8-Lip	Breast cancer and liver cancer	Effective immune evasion and tumor enrichment	Nanoparticle hydrated size increased to ~ 99.6 nm	[153]
TPL-GNR@MSN-3P	Cancer	TPL-GNR@MSN-3P accumulated at the tumor site within 6 h	Drug release was triggered only upon concurrent high intracellular GSH and external laser irradiation	[140]
TPL-NPs	Chronic dermatitis	Transdermal permeation was significantly enhanced	Sudden release occurred after 12 h	[139]
D-Trip	Glioblastoma	D-Trip reduced tumor burden to 12% (from 35%) and cut liver toxicity fivefold in a glioblastoma model	Only approximately 30% TP was released within 24 h	[143]
(SFN + TPL)@CPLCNPs	Hepatocellular carcinoma	69.4% apoptosis in Huh-7 cells, superior in vivo tumor suppression, prolonged circulation without aggregation	Initial rapid release phenomenon	[131]
FA + TPP-TP-Lips	Hepatocellular carcinoma	79.37% tumor inhibition rate in tumor-bearing mice	In vivo AST elevation implied hepatotoxic risk	[145]
NF-Trip	Hepatocellular carcinoma	NF-Trip increased survival to 80% with reduced toxicity in mice, compared to 30% in controls	Drug release depended on folate receptor expression and an acidic environment	[128]
TP/GLLNP	Hepatocellular carcinoma	Effective tumor inhibition (87.2%) with low hemolysis (< 5%) and minimal systemic toxicity at therapeutic doses	Slow drug release and liver accumulation	[151]
TF-TP@LIP	Hepatocellular carcinoma	Potent tumor-targeted activity (IC_50_ = 42.3 nM)	Long-term stability required further validation	[141]
TPL@mPLGA	Hepatocellular carcinoma	The tumor growth inhibition rate reached as high as 69.25%	Slow release hindered rapid drug action at the tumor	[150]
PSSP@TP	Hepatocellular carcinoma	PSSP@TP significantly enhanced the therapeutic index of TP by boosting antitumor efficacy while reducing hepatotoxicity in LO2 cells	Low encapsulation efficiency	[133]
TPL@TFBF	Melanoma	TPL@TFBF achieved a 65.6% anti-tumor rate with a hemolysis rate below 2%, indicating high safety	Drug efficacy depended on an acidic microenvironment	[152]
TPL@PLGA@F127	Myocardial infarction	Cardiac function was effectively improved while reducing toxicity	Burst release and poor myocardial retention	[142]
TP-TPBC-PEG	Osteosarcoma	Mortality from 60% (free TP) to 0% (nanomedicine); severe multi-organ damage prevented	Long-term safety and in vivo metabolic profile required further validation	[124]
PVGLIG-MTX-D/T-NMs	Ovarian cancer	PVGLIG-MTX-D/T-NMs achieved a tumor suppression rate exceeding 50%	Particle size stability was below the ideal level	[160]
TP-SP@NPs	Pancreatic cancer	Targeting both pancreatic cancer cells (IC_50_ 14.62 ng/mL) and M2 macrophages (IC_50_ 7.55 ng/mL)	Stability in certain biological media was limited	[129]
TPL-LA-lip	Pancreatic cancer	The survival period of tumor-bearing mice was extended from 28 to 45 days	The prodrug (TPL-LA) was metabolized slowly in blood plasma, with 32.68% converted after 24 h	[191]
NVs	Pancreatic cancer	A 10.6-fold increase in tumor accumulation, M2-to-M1 macrophage repolarization, and 56% tumor cell apoptosis with low systemic toxicity	The blood half-life of NVs was 17.75 h, and risks elevated liver and spleen accumulation	[154]
Sequential delivery (CRE-NP(α-M) + CRP-MC(Trip))	Pancreatic ductal adenocarcinoma	Median survival was significantly extended to 50 days (vs. 28 days in controls)	The complexity of the delivery system posed challenges for clinical translation and large-scale production	[158]
T10-AHNAK-MBs	Parkinson’s disease	T10-AHNAK-MBs enhanced brain drug delivery via ultrasound, reducing neuron loss to 21.4%, restoring dopamine to 414.1 ng/mL, and improving motor function in Parkinson’s mice	This microbubble system required focused ultrasound to open the blood–brain barrier	[99]
TP/BIBF-bHDL	Renal fibrosis	TP/BIBF-bHDL markedly improved kidney injury targeting while reducing systemic toxicity, with LD_50_ increased from 0.48 to 0.88 mg/kg	Long-term distribution within the body was influenced by hepatic uptake	[157]
TP-FPNPs	Renal ischemia–reperfusion injury	A 3.2-fold higher drug accumulation in the kidneys, a 2.9-fold lower acute tubular injury score, and alleviated off-target toxicity	Relatively low loading capacity (1.11%)	[126]
TP@NPs	Rheumatoid arthritis	Substantially lower toxicity than free TP	Off-target risk persists due to premature drug release (~ 21% within 36 h)	[125]
FA-TP@VA NPs	Rheumatoid arthritis	FA-TP@VA NPs showed approximately 3 times higher cellular uptake than unmodified nanoparticles	Notable off-target accumulation in the liver and spleen	[146]
PAT	Rheumatoid arthritis	High drug loading (48.6%) and sustained release (29 h half-time), combined with reduced toxicity of free TP	In vitro, the toxicity of PAT converged with that of the free TP over a 72-h exposure period	[147]
TP-LCNPS-GEL	Rheumatoid arthritis	TP-LCNPS-GEL effectively reduced organ toxicities caused by oral administration	Its phase transition occured at approximately 32°C, near body temperature	[135]
TP/PNPs	Rheumatoid arthritis	TP/PNPs targeted macrophages via cluster of differentiation 44, alleviating arthritis symptoms in rats with reduced systemic toxicity	Drug release was slow in a neutral environment	[153]
TPL-NLC	Rheumatoid arthritis	TPL-NLC significantly alleviated knee joint swelling and lowered inflammatory cytokine levels in rheumatoid arthritis rats	Once-daily dosing for 14 consecutive days to maintain therapeutic efficacy	[134]
GDR-TPT	Rheumatoid arthritis	GDR-TPT effectively targeted and accumulated in inflamed joints	Less than 20% drug was released after 48 h in a pH 7.4 environment	[144]
HTC@ZIF8	Triple-negative breast cancer	HTC@ZIF8 enhanced tumor platinum delivery by twofold and increased normal cell IC_50_ by over 1700 times, improving both efficacy and safety	HTC@ZIF8’ large hydrated size (~ 261 nm) hindered deep tumor penetration and uniform distribution	[159]
(TP + A)@TkPEG NPs	Triple-negative breast cancer	(TP + A)@TkPEG achieved light-triggered drug release (90.9%), extending survival to 60 days in 40% of mice, surpassing the fully lethal control	Therapeutic efficacy was highly dependent on external light activation	[132]

TP: Triptolide; GSH: Glutathione

### Nanotechnology-based strategies to mitigate toxicity of triptolide

The clinical application of triptolide has been primarily limited by its severe systemic toxicity. Conventional formulations have led to rapid biodistribution, resulting in widespread tissue damage [14, 15]. Fortunately, nanotechnology has offered promising strategies to mitigate these challenges. By enabling targeted delivery, enhancing cellular uptake, and improving site-specific accumulation, nanoscale formulations have significantly reduced the off-target toxicity of triptolide while maintaining therapeutic efficacy [126, 128, 142, 143].

#### Stimuli-responsive nanoplatforms for triptolide delivery

Triptolide-based smart delivery systems have demonstrated significant potential in improving drug safety by responding to specific microenvironments [144]. For glioblastoma treatment, dendrimer-triptolide conjugates selectively targeted tumor-associated macrophages, induced intracellular drug release and reprogrammed macrophages from a pro-tumor to an anti-tumor phenotype, thereby enhancing therapeutic effects while markedly reducing hepatotoxicity and cardiotoxicity [143].

TP-TPBC-PEG nanospheres enabled controlled triptolide release through dual pH- and near-infrared-responsive mechanisms, activating Hippo pathway to induce tumor cell death. Notably, the nanospheres exhibited no organ toxicity in mice, suggesting promise for both primary osteosarcoma treatment and lung metastasis prevention [124]. For rheumatoid arthritis, pH-sensitive TP@NPs selectively accumulated in the acidic synovial microenvironment to deliver potent anti-inflammatory and chondroprotective effects at ultra-low doses, effectively minimizing systemic exposure and overcoming the toxicity limitations of free triptolide for safer therapeutic outcomes [125].

#### Targeted delivery strategies for enhanced triptolide nanomedicine accumulation

Targeted optimization of triptolide nanomedicines has significantly enhanced tumor-specific drug accumulation while reducing off-target distribution, thereby lowering systemic toxicity [128]. Sulfated dextran and arginine-glycine-aspartate peptide co-modified TP-SP@NPs demonstrated efficient tumor accumulation and potent anti-pancreatic cancer activity by reducing pro-tumor M2 macrophage infiltration, with significantly lower systemic toxicity than free triptolide, showing strong clinical translation potential [129]. Furthermore, mitochondrial-targeted triptolide liposomes FA + TPP–TP–Lips specifically localized to liver cancer cell mitochondria, disrupted membrane potential and elevated intracellular ROS levels, significantly increased tumor cell apoptosis while reducing hepatotoxicity and systemic side effects, establishing a promising targeted strategy for hepatocellular carcinoma therapy with enhanced efficacy and reduced toxicity [145].

For rheumatoid arthritis treatment, FA-TP@VA nanoparticles achieved targeted delivery and efficient accumulation in inflamed joints through folate receptor-mediated uptake by M1 macrophages, significantly enhancing therapeutic efficacy while reducing triptolide-induced hepatic oxidative stress and systemic toxicity [146]. Similarly, PAT nanocarriers selectively accumulated in affected joints, showing comparable therapeutic efficacy to free triptolide while exhibiting markedly reduced toxicity, thus offering a safer treatment option for rheumatoid arthritis [147].

#### Cellular-uptake boosted triptolide nanomedicine

The cellular uptake efficiency of triptolide nanomedicines has been significantly enhanced by ligand modification. For instance, folate-functionalized Pluronic F127/P123 nanoparticles encapsulating triptolide facilitated folate receptor-mediated endocytosis, improving drug delivery for renal ischemia–reperfusion injury while reducing renal, hepatic, and reproductive toxicity, thereby demonstrating superior therapeutic safety [126]. Similarly, hyaluronic acid-coated triptolide nanomedicines promoted targeted accumulation in breast cancer tissue, enhancing antitumor efficacy and minimizing systemic toxicity [127].

### Nanotechnology-based strategies to enhance efficacy of triptolide

The nanodelivery system for triptolide has employed multidimensional strategies, including targeted drug delivery, combination therapy, tumor microenvironment modulation, and pharmacokinetic optimization, to significantly enhance drug accumulation and therapeutic efficacy at disease sites [148]. These innovations have substantially improved the clinical potential of triptolide in treating cancers, rheumatoid arthritis, and chronic kidney disease [148, 149].

#### Active-targeting nanodelivery system for site-specific triptolide accumulation

In tumor therapy, innovative nanocarrier systems have demonstrated remarkable targeting precision and therapeutic potential. Transferrin-modified triptolide liposomes TF-TP@LIP exploited transferrin receptor-mediated endocytosis to enhance drug accumulation in hepatocellular carcinoma tissues, significantly improving antitumor efficacy [141]. Biomimetic TPL@mPLGA nanoparticles, functionalized with Huh-7 cell membranes, achieved homologous tumor targeting, promoting active drug deposition and demonstrating potent anti-hepatocellular carcinoma activity in both in vitro and in vivo models [150]. Additionally, glycyrrhizic acid-based lipid nanoparticles TP/GLLNP not only enhanced triptolide uptake, cytotoxicity, and apoptosis in HepG2 cells but also exhibited superior tumor retention in H22 xenografts, offering a novel strategy for synergistic hepatocellular carcinoma therapy [151].

Similarly, DT/Pep1 system achieved targeted co-delivery of doxorubicin and triptolide to breast cancer through peptide-mediated recognition, and exploited nanoparticle morphology transitions to prolong tumor retention, significantly enhancing intracellular drug accumulation and apoptotic induction, representing an effective and safe therapeutic approach for breast cancer [138]. TPL@TFBF employed fatty acid-mediated cellular uptake to co-deliver triptolide, Fe^3^⁺, and tannic acid into tumor cells, where it triggered a cascade of therapeutic effects including Fenton reaction-driven ROS generation, Nrf2 pathway inhibition, dual induction of ferroptosis (via GPX4 inactivation) and pyroptosis (through Caspase-3/GSDME activation), while concurrently activating dendritic cell-mediated antitumor immunity, establishing a novel multimodal immunogenic cell death strategy for melanoma therapy [152].

In the treatment of inflammatory diseases, cluster of differentiation 44 receptor-targeted polymeric nanoparticles TP/PNPs leveraged the “ELVIS” effect and macrophage-selective uptake mechanisms to achieve efficient triptolide delivery to arthritic lesions, while their pH-responsive design facilitated lysosomal escape to further enhance inflammatory site-specific drug accumulation, ultimately providing effective symptomatic relief in rheumatoid arthritis [153].

#### Co-encapsulated triptolide nanomedicines for combination therapy

Triptolide-based nanocarriers have provided an ideal platform for combination therapy by co-delivering multiple drugs with complementary mechanisms of action. This strategy has enhanced synergistic effects, reduced drug resistance, and lowered individual drug doses [154]. PTPP was a tumor-targeting nanomedicine co-delivering paclitaxel and triptolide, exhibiting excellent solubility and synergistic therapeutic effects. By modulating NF-κB pathway, it induced ROS generation and enhanced ferroptosis, overcame chemotherapy resistance effectively, providing a promising approach for treating non-small cell lung cancer [130]. The bio-inspired nanomedicine system (SFN + TPL)@CPLCNPs, which simultaneously delivered sorafenib and triptolide for tumor-targeted therapy, not only inhibited liver tumor growth and promoted apoptosis but also exhibited strong synergistic effects with reduced toxicity, presenting a clinically viable solution for hepatocellular carcinoma treatment [131].

In breast cancer treatment, triptolide-based nanocarriers have exhibited synergistic therapeutic effects through multimodal mechanisms. The red blood cell membrane-coated, R8-modified biomimetic liposomes Cel + TP/RBCm@R8-Lip evaded reticuloendothelial clearance and achieved tumor-specific delivery via R8-mediated endocytosis, where the released triptolide and celastrol simultaneously induced apoptosis, suppressed metastasis/invasion, and disrupted autophagy in breast and liver cancers, offering an efficient combination therapy [155]. Additionally, triptolide-naringin conjugate nanoparticles enabled EGFR-targeted delivery while concurrently inhibiting both EGFR and PI3K/AKT pathways, presenting a novel natural product-based targeted therapy for breast cancer [156].

Triptolide nanocarriers have also delivered effective treatment for non-tumor diseases through synergistic drug combinations. Biomimetic high-density lipoprotein nanoparticles co-encapsulating triptolide and nintedanib actively remodeled fibrotic microenvironments by concurrently suppressing inflammatory cytokines, blocking immune cell infiltration, and preventing myofibroblast activation, achieving significant reduction in renal fibrosis and opening new therapeutic avenues for chronic kidney disease [157].

#### Exploiting aberrant tumor microenvironment features for smart triptolide nanodelivery

The pathological features of the tumor microenvironment, including hypoxia, acidic pH, elevated GSH levels, and immune suppression, create both therapeutic barriers and opportunities for smart drug delivery system design [148]. Emerging microenvironment-responsive nanosystems have significantly advanced antitumor potential of triptolide [158]. The (TP + A)@TkPEG system, which integrated a photosensitizer, an aggregation-induced emission material, and autophagy-regulator triptolide within ROS-responsive nanoparticles, amplified ROS and controlled triptolide release in 4T1 cells, concurrently inducing apoptosis and suppressing Beclin-1-mediated autophagy, establishing a novel therapeutic paradigm for triple-negative breast cancer [132].

GSH-sensitive triptolide-loaded nanoparticles have achieved sustained drug delivery through a GSH-triggered mechanism, significantly enhancing tumor-specific drug accumulation and antitumor efficacy [133]. The temperature- and redox-responsive nanocarrier TPL-GNR@MSN-3P enabled precise triptolide release under high intratumoral GSH levels and localized near-infrared-induced heating, effectively counteracting the heat shock response from photothermal therapy to reduce cancer cell thermal tolerance [140]. Similarly, the acid- and GSH-responsive nanoplatform HTC@ZIF8 depleted GSH and inhibited GPX4 activity in the tumor microenvironment, thereby synergizing with cisplatin while releasing triptolide, which further suppressed GSH synthesis via Nrf2 regulation to induce dual apoptosis and ferroptosis in breast cancer cells, offering a promising clinical translation strategy for breast cancer therapy [159].

Additionally, PVGLIG-MTX-D/T-NMs released docetaxel and triptolide in response to high levels of matrix metalloproteinase-2 in the tumor microenvironment, which enhanced tumor cell uptake of the drugs and inhibited tumor angiogenesis and metastasis by regulating the expression of proteins related to epithelial-mesenchymal transition, presenting a new approach for targeted ovarian cancer therapy [160].

#### Triptolide-loaded nanocarriers with optimized pharmacokinetic profiles

Delivery systems including nanolipid carriers, cubic and hexagonal liquid crystals, and C16-N nanofibers have significantly improved the transdermal penetration and sustained release of triptolide, demonstrating strong potential for treating rheumatoid arthritis and hepatocellular carcinoma [134–137].

Triptolide-loaded nanostructured lipid carriers TPL-NLCs demonstrated excellent drug permeability and sustained-release properties, effectively enhancing transdermal drug absorption while maintaining high local drug concentrations, thereby offering a promising delivery platform for transdermal rheumatoid arthritis therapy [134].

Triptolide-loaded cubic and hexagonal liquid crystals significantly improved transdermal drug delivery by enhancing the 48 h cumulative skin penetration, achieving higher drug concentrations in the skin than in the bloodstream, thereby markedly increasing bioavailability. These systems exerted anti-inflammatory effects by reducing TNF-α and IL-1β levels, effectively alleviating foot swelling in arthritic rats and suppressing inflammatory responses, which confirmed the therapeutic potential of triptolide for rheumatoid arthritis treatment [135, 136].

Furthermore, the triptolide-loaded C16-N nanofibers achieved sustained drug release for 14 days, showing remarkable therapeutic effects against hepatocellular carcinoma. After intraperitoneal injection of the C16-N/T hydrogel, the drug preferentially accumulated and persisted in mouse liver tissue for 13 days, significantly suppressing in situ tumor growth and extending the median survival time of tumor-bearing mice by twofold, underscoring the potential of triptolide for hepatocellular carcinoma chemotherapy and combination therapies [137].

## Future perspectives and translational potential

Considerable studies have explored and confirmed that triptolide exerted pleiotropic pharmacological activities, such as antitumor, anti-inflammatory, and immunomodulatory effects, by regulating multiple types of programmed cell death [161, 162]. However, its clinical application has remained encumbered by challenges, including potential severe hepatotoxicity, nephrotoxicity, cardiotoxicity, and reproductive toxicity [163–165]. Future research should concentrate on increasing organ targeting, alleviating toxicities, identifying precise molecular targets of triptolide, and developing innovative delivery systems based on nanocarriers [139, 159, 166, 167].

Among the various delivery strategies for triptolide, engineered systems that can “sense” pathological cues, “navigate” to target tissues, and “activate” at disease sites while orchestrating multiple mechanisms to address complex disorders have been a focal point of contemporary research [143, 144]. These approaches have demonstrated promising efficacy and favorable biosafety across a range of preclinical models [168]. Such advances are expected to markedly enhance the targeting efficiency of triptolide, minimize systemic toxicity, and improve therapeutic outcomes, thereby offering crucial support for its clinical safety and efficacy [125, 139, 143].

### Elucidating the molecular targets of triptolide

The reported direct targets of triptolide majorly include Xeroderma Pigmentosum group B, TGF-beta activated kinase 1 binding protein 1 (TAB1), XIAP, and HSP70, among others [119, 169–173]. Specifically, triptolide have covalently modified the Cys342 residue of Xeroderma Pigmentosum group B [169], subsequently inducing degradation of the RNA polymerase II core subunit Rpb1 [170], thus exerting anticancer and immunosuppressive effects [171]; triptolide has bound to TAB1 and interfering with TAK1-TAB1 complex formation, resulting in inhibition of TAK1 kinase activity and anti-inflammatory effects [172]; triptolide has directly targeted XIAP to modulate the XIAP/COMMD1/ATP7A/B signaling axis, disrupting intracellular copper homeostasis and inducing cuproptosis in cervical cancer cells [119]; and triptolide has inhibited HSP70 activity, affecting cellular stress responses and protein folding processes, supporting antitumor and immunomodulatory functions [173].

Beyond the validated targets discussed, a number of potential targets currently remain at the stage of in silico prediction or functional correlation, necessitating further validation through affinity techniques. Song et al. [174] combined network pharmacology with molecular docking to predict six potential target proteins (MCL-1, MAPK8, CXCL8, and STAT1) for triptolide in rheumatoid arthritis treatment, providing direction for subsequent mechanism studies. Liang et al. [5] utilized colorectal cancer mice, organoids, cell lines, and clinical samples to ascertain that triptolide inhibited RNA polymerase III transcription by blocking the TBP/TFIIB-related factor 1 interaction, thereby reduced TFIIIB formation on tRNA and 5S rRNA promoters, offering a novel mechanism for treating colorectal cancer.

### Conducting scientifically rigorous clinical trials

Triptolide has been observed to exert activities and toxicities on the liver, kidneys, and heart, demonstrating a protective organ effect at low doses but inducing organ toxicities at high doses [12]. Hepatotoxicity is the most prevalent and clinically significant risk associated with the use of triptolide preparations, characterized by complex mechanisms and insidious development [14]. In clinical practice, regular monitoring of serum markers, including alanine aminotransferase, aspartate aminotransferase, and total bilirubin, is essential. Based on the observed adverse events and laboratory results, appropriate dose adjustments should be implemented to manage potential toxicity of triptolide and triptolide preparations [175].

Moreover, the tested concentration ranges in bioassay applied in current in vitro studies have varied widely, such as from 0.01 to 1 nM [41, 43], which may prevent experimental results from accurately reflecting the true biological effects of triptolide, thereby undermining clinical translational value. Consequently, these limitations have underscored the necessity for further validation of triptolide’s efficacy, safety, and pharmacokinetic profile in humans through rigorously designed clinical trials. To date, this review has collected 13 registered clinical trials involving triptolide (and its formulations and derivatives) via searches of the International Clinical Trials Registry Platform, ClinicalTrials.gov, Chinese Clinical Trial Registry, and the Drug Clinical Trial Registration and Information Disclosure Platform with details summarized in Table 4. Notably, two of these trials have reported key outcomes. A Phase 2 study of (5R)-5-hydroxytriptolide (LLDT-8) with long-term suppressed but immunologically non-responsive HIV/AIDS has indicated that oral administration of LLDT-8 (0.5 mg or 1 mg once daily for 48 weeks) effectively promoted CD4^+^ T-cell recovery and reduced inflammation [176]. Additionally, a Phase 3 trial of a triptolide-containing formulation with autosomal dominant polycystic kidney disease has demonstrated that oral administration (1 mg/kg per day, dynamically adjusted over 6 months) significantly reduced proteinuria from 2645 ± 1408 mg/d to 702 ± 418 mg/d post-treatment [175].

**Table 4:** Clinical progress of triptolide formulations/derivatives

Derivatives/formulations	Indication	Phases	Intervention	Key outcomes	TrialID
(5R)-5-hydroxytriptolide (LLDT-8)	HIV/​AIDS	Phase 2 (Completed)	Oral LLDT-8 0.5 mg or 1 mg daily for 48 weeks	LLDT-8 demonstrated potential as a therapeutic option by enhancing CD4 recovery and reducing inflammation in long-term suppressed immunological non-responders	NCT04084444
Glucosidorum Tripterygll Totorum	Autosomal dominant polycystic kidney disease	Post-market (Not Recruiting)	Glucosidorum Tripterygll Totorum + losartan potassium	Unknown	ChiCTR-TRC-11001282
Minnelide Capsules	Advanced solid tumors	Phase 1 (Recruiting)	Minnelide Capsules administered orally once daily for 21 days followed by a 7‑day break, with or without combination intravenous protein‑bound paclitaxel on days 1, 8, and 15 in pancreas and breast cancer patients, in 28‑day cycles	Unknown	NCT03129139
Minnelide	Non-small cell lung cancer	Phase 1b (Recruiting)	Minnelide administered orally once daily on days 1‑21 and osimertinib orally once daily on days 1‑28. Cycles repeat every 28 days for 6 months	Unknown	NCT05166616
Minnelide	Adenosquamous carcinoma of the pancreas	Phase 2 (Completed)	Minnelide administered orally (2 mg) once daily for 21 days of 28‑day cycles for 12 cycles	Unknown	NCT04896073
Tripterygium glycosides	Crohn’s disease	Phase 2/Phase 3 (Unknown)	Mesalazine 4 g/day and Tripterygium glycosides 2 mg/kg/day for 12 weeks	Unknown	NCT02044952
Tripterygium wilfordii granules	Psoriasis	N/A (Recruiting)	Tripterygium wilfordii granules	Unknown	ChiCTR2000036398
Triptolide Woldifii	HIV	Phase 1/Phase 2 (Recruiting)	Triptolide 2 tablets three times daily orally (20 mg per dose) for 12 months, in combination with cART	Unknown	NCT01817283
Triptolide Woldifii	HIV /AIDS	Phase 3 (Completed)	Triptolide Wilfordii 20 mg three times daily orally combined with ART for 12 months	Unknown	NCT03403569
Triptolide Woldifii	Polycystic Kidney	N/A (Terminated)	Triptolide Woldifii 60 mg/day	Unknown	NCT00801268
Triptolide Woldifiion	HIV /AIDS	Phase 3 (Recruiting)	Triplitode 2 tablets three times daily orally for 12 months after initial 6 months of cART	Unknown	NCT02219672
Triptolide-Containing Formulation	Autosomal dominant polycystic kidney disease	Phase 3 (Recruiting)	Triptolide‑Containing Formulation 1 mg/kg/day orally in three divided doses, with dose adjustment based on adverse events monitoring, for a core treatment period of 6 months	After 6 months of Tripterygium therapy, a reduction of proteinuria from 2,645 ± 1,408 mg/d to 702 ± 418 mg/d	NCT02115659
Tripterygium Wilfordii Hook F extract	HIV	N/A (Completed)	Tripterygium Wilfordii Hook F extract 10 mg three times daily orally plus current cART	Unknown	NCT02002286

LLDT-8: (5R)-5-hydroxytriptolide; HIV: Human Immunodeficiency Virus; AIDS: Acquired Immunodeficiency Syndrome; cART: combination Antiretroviral Therapy; ART: Antiretroviral Therapy; N/A: Not Applicable

In the future, well-designed, large-scale, multicenter randomized controlled trials are needed to systematically evaluate triptolide’s efficacy and safety across different indications, clarify its specific plasma concentrations corresponding to the therapeutic and toxic windows, dose/concentration-effect relationship, and potential adverse reactions from long-term use. Such studies would provide more robust evidence-based medical support for the clinical application of triptolide.

## Concluding remarks

Triptolide has been demonstrated to regulate a variety of programmed cell death processes, including apoptosis, autophagy, pyroptosis, ferroptosis, cuproptosis, necroptosis, and PANoptosis, through a complex and precise network of signaling pathways. It has shown considerable potential in the treatment of cancer, immune and inflammatory diseases, and neurodegenerative diseases, but its clinical translation has been severely limited by hepatotoxicity, nephrotoxicity, cardiotoxicity, and reproductive toxicity. To overcome these limitations, advanced nano-delivery systems offer a promising strategy to enhance the targeted delivery and controlled release of triptolide, thereby reducing its toxicity while improving therapeutic efficacy. In the future, clarifying triptolide’s precise target mechanisms and conducting internationally compliant clinical trials are anticipated to overcome the clinical application limitations of triptolide and promote it as a highly effective and low-toxicity candidate drug for clinical use. This review provides theoretical references for the clinical translation of triptolide.

## Data Availability

No datasets were generated or analysed during the current study.

## Abbreviations

ACER1: Alkaline ceramidase 1
AIDS: Acquired immunodeficiency syndrome
AKT: AKT serine/threonine kinase
AML: Acute Myeloid Leukemia
AMPK: AMP-activated protein kinase
ART: Antiretroviral therapy
ATF4: Activating transcription factor 4
ATG5: Autophagy related 5
ATP7A: ATPase copper transporting alpha
BAD: Bcl-2-associated death promoter
BAX: BCL2-associated X protein
BCL-2: B-cell lymphoma 2
CARD9: Caspase recruitment domain-containing protein 9
cART: Combination antiretroviral therapy
CASP10: Caspase-10
CASP8: Caspase-8
CFLAR: Cellular FLICE-like inhibitory protein
CHOP: C/EBP homologous protein
CML: Chronic Myeloid Leukaemia
c-Myc: Cellular myelocytomatosis viral oncogene homolog
COMMD1: Copper metabolism domain containing 1
CX3CR1: C-X3-C motif chemokine receptor 1
CXCL: C-X-C motif chemokine ligand
Drp1: Dynamin-related protein 1
ERK: Extracellular signal-regulated kinase
Fas: Fas cell surface death receptor
FLIP: FLICE-like inhibitory protein
FTH1: Ferritin heavy chain 1
Fyn: FYN proto-oncogene, Src family tyrosine kinase
GPX4: Glutathione peroxidase 4
GRP78: Glucose-regulated protein 78
GSDME: Gasdermin E
GSH: Glutathione
GSK3β: Glycogen synthase kinase-3 beta
HIV: Human Immunodeficiency Virus
HKII: Hexokinase II
HO-1: Heme oxygenase 1
HSP70: Heat shock protein 70
i.g.: Intragastric administration
i.p.: Intraperitoneal injection
IFN-γ: Interferon-gamma
IgA: Immunoglobulin A
IL: Interleukin
NK: C-jun N-terminal kinase
Keap1: Kelch-like ECH-associated protein 1
K63: Lysine 63
LC3: Microtubule-associated protein 1 light chain 3
LLDT-8: (5R)-5-hydroxytriptolide
LPS: Lipopolysaccharide
MAPK: Mitogen-activated protein kinase
MCL-1: Myeloid cell leukemia 1
MDA: Malondialdehyde
miR-221: MicroRNA-221
MLKL: Mixed lineage kinase domain-like
mTOR: Mechanistic target of rapamycin
N/A: Not applicable
NF-κB: Nuclear Factor kappa-light-chain-enhancer of activated B cells
NLRP3: NOD-like receptor family, pyrin domain-containing 3
NRF1: Nuclear respiratory factor 1
Nrf2: Nuclear factor erythroid 2-related factor 2
NSCLS: Non-small cell lung cancer
PARP: Poly(ADP-ribose) polymerase
PCD: Programmed cell death
PFKFB2: Phosphofructo-2-kinase/fructose-2,6-bisphosphatase 2
PGC-1α: Peroxisome proliferator-activated receptor gamma coactivator 1-alpha
PI3K: Phosphatidylinositol 3-kinase
PPIL3: Peptidylprolyl isomerase like 3
PTEN: Phosphatase and Tensin homolog deleted on chromosome 10
PUMA: P53 upregulated modulator of apoptosis
Rab7: RAS-associated protein RAB-7
RIPK: Receptor-interacting protein kinase
ROS: Reactive oxygen species
s.c.: Subcutaneous injection
SIRT1: Sirtuin 1
SLC7A11: Solute carrier family 7 member 11
SRC: Sarcoma proto-oncogene, non-receptor tyrosine kinase
STAT: Signal transducer and activator of transcription3
TAB1: TGF-beta activated kinase 1 binding protein 1
TBK1: TANK-binding kinase 1
TF: Transferrin
TFR1: Transferrin receptor 1
TNF-α: Tumor necrosis factor-alpha
TP: Triptolide
TRAIL: Tumor necrosis factor-related apoptosis-inducing ligand
Wnt: Wingless/integrated
XIAP: X-linked inhibitor of apoptosis protein
